# Tomato FK506 Binding Protein 12KD (FKBP12) Mediates the Interaction between Rapamycin and Target of Rapamycin (TOR)

**DOI:** 10.3389/fpls.2016.01746

**Published:** 2016-11-18

**Authors:** Fangjie Xiong, Pan Dong, Mei Liu, Gengxin Xie, Kai Wang, Fengping Zhuo, Li Feng, Lu Yang, Zhengguo Li, Maozhi Ren

**Affiliations:** ^1^School of Life Sciences, Chongqing UniversityChongqing, China; ^2^Center of Space Exploration, Ministry of EducationChongqing, China

**Keywords:** tomato, target of rapamycin, SlFKBP12, rapamycin, KU63794, gene expression profile

## Abstract

Target of Rapamycin (TOR) signaling is an important regulator in multiple organisms including yeast, plants, and animals. However, the TOR signaling in plants is much less understood as compared to that in yeast and animals. TOR kinase can be efficiently suppressed by rapamycin in the presence of functional FK506 Binding Protein 12 KD (FKBP12) in yeast and animals. In most examined higher plants rapamycin fails to inhibit TOR kinase due to the non-functional FKBP12. Here we find that tomato plants showed obvious growth inhibition when treated with rapamycin and the inhibitory phenotype is similar to suppression of TOR causing by active-site TOR inhibitors (asTORis) such as KU63794, AZD8055, and Torin1. The chemical genetic assays using TOR inhibitors and heterologous expressing SlFKBP12 in *Arabidopsis* indicated that the TOR signaling is functional in tomato. The protein gel shifting and TOR inhibitors combination assays showed that SlFKBP12 can mediate the interaction between rapamycin and TOR. Furthermore, comparative expression profile analysis between treatments with rapamycin and KU63794 identified highly overlapped Differentially Expressed Genes (DEGs) which are involved in many anabolic and catabolic processes, such as photosynthesis, cell wall restructuring, and senescence in tomato. These observations suggest that SlFFBP12 is functional in tomato. The results provided basic information of TOR signaling in tomato, and also some new insights into how TOR controls plant growth and development through reprogramming the transcription profiles.

## Introduction

Target of Rapamycin (TOR) is a Ser/Thr protein kinase that was first discovered in budding yeast (*Saccharonmyces cerevisiae*) by a genetic selection for screening the mutants which are insensitive to rapamycin (Heitman et al., 1991). Rapamycin, known as sirolimus, a secondary metabolite produced by the soil bacteria *Streptomyces hygroscopicus*, specifically binds to FK506 Binding Protein 12 KD (FKBP12) and forming a binary complex. The rapamycin-FKBP12 complex further binds to FRB domain of TOR to form rapamycin-FKBP12-TOR ternary complex, resulting in partial abolishment of TOR kinase activity (Heitman et al., 1991; Chiu et al., 1994; Sabatini et al., 1994). Based on the rapamycin-FKBP12-TOR system, TOR and its signaling pathway have been extensively studied in yeast and mammals. The accumulated documents showed that TOR is a central coordinator of energy, nutrient, and stress signaling networks from yeast to mammals and plants (Henriques et al., 2014; Rexin et al., 2015; Xiong and Sheen, 2015; Dobrenel et al., 2016). Five highly conserved domains: HEAT repeats, FAT, FRB, kinase, and FATC reside in TOR protein from N-terminal to C-terminal (Henriques et al., 2014; Rexin et al., 2015). Most eukaryotic organisms contain one copy of TOR gene, but two and three TOR genes were identified in yeast and *Leishmania major*, respectively (Heitman et al., 1991; Crespo et al., 2005; Madeira da Silva et al., 2009; Cornu et al., 2013). The TOR protein recruits RAPTOR (regulatory-associated protein of TOR) and LST8 (lethal with SEC13 protein 8) to form TOR complex 1 (TORC1), and combines LST8, SIN1 (stress-activated map kinase-interacting protein 1) and RICTOR (rapamycin insensitive companion of TOR) to form the TOR complex 2 (TORC2) in yeast and animals (Lee et al., 2005). Rapamycin-sensitive TORC1 plays a major role in cell growth, development, and proliferation in a temporal manner, while rapamycin-resistant TORC2 seems to spacially regulate the development of cell cytoskeleton (Loewith et al., 2002; Wang and Proud, 2009; Takahara and Maeda, 2013; Xiong and Sheen, 2014).

The peptidyl prolil isomerase FKBP12 is a key player to bridge the interaction between rapamycin and TOR. The mutations in the FKBP12 gene result in rapamycin insensitivity (Koltin et al., 1991). Most plants such as *Arabidopsis thaliana, Vicia faba, Oryza Sativa, Nicotiana tabacum*, and *Brassica napus*, etc., are insensitive to rapamycin (Xu et al., 1998; Menand et al., 2002; Ren et al., 2012). Rapamycin belongs to the first generation of TOR inhibitor. The rapamycin insensitivity of most examined plants largely limited our understanding of TOR signaling in plants. However, recent studies showed that, active-site TOR inhibitors (asTORis), the second generation TOR inhibitors, were successfully applied to decipher TOR signaling in plants (Montané and Menand, 2013; Xiong and Sheen, 2014; Dong et al., 2015; Xiong et al., 2016). More than 30 different asTORis have been developed (Montané and Menand, 2013). The structures of these asTORis are different, but they can directly bind to the kinase domain of TOR by competing with ATP. KU63794, TORIN1, and AZD8055 represent mild, moderate and strong asTORis with high specificity against TOR kinase domain in animals and plants (Montané and Menand, 2013; Xiong et al., 2016).

In this study, we found that the growth of tomato plants was retarded by rapamycin, indicating that SlFKBP12 may be functional in tomato. This hypothesis was further confirmed by rapamycin susceptibility restore assays in *Arabidopsis* expressing *SlFKBP12*. The protein gel shifting assay and TOR inhibitors combination assays showed that SlFKBP12 can mediate the interaction between rapamycin and TOR. Additionally, RNA Sequencing (RNA-Seq) experiments were performed to examine the gene expression profiling of tomato seedlings treated with rapamycin and KU63794, respectively. The huge amount of Differentially Expression Genes (DEGs) involving in many anabolic and catabolic processes, such as photosynthesis, cell wall restructuring, and senescence were observed in rapamycin or KU63794 treated tomato. Importantly, the data shows rapamycin and KU63794 can generate highly overlapping gene expression profiling in tomato. These independent evidences suggest that SlFKBP12 play key role in mediating the interaction between rapamycin and TOR signaling in tomato.

## Materials and methods

### Tomato growth conditions and measurements

Tomato (*Solanum lycopersicum*, cv. Micro Tom) seeds were sterilized with 70% ethanol for 2 min, followed by 10% sodium hypochlorite for 10 min, and then washed 5–6 times with distilled water. The surface-sterilized seeds were germinated on square Petri dishes (100 × 100 × 17 mm) with half-strength Murashige–Skoog (0.5 × MS) agar supplemented with different inhibitors (rapamycin, AZD8055, TORIN1, and KU63794, selleckchem) at increasing concentrations (0, 1, 5, and 10 μM). The seedlings were photographed and primary root length and fresh weight were measured at 7 days after germination (DAG), respectively. In addition, normal seedlings of 5DAG were transferred to 0.5 × MS medium on Petri dishes with 10 μM different inhibitors (rapamycin, AZD8055, TORIN1, and KU63794) for 5 more days' growth. Then, the fresh weight and root length were measured. All plants were grown in growth chambers at 25°C and a photoperiod of 16 h (100-mmol/m^2^/s fluorescence bulb light) followed by 8 h of dark unless indicated otherwise.

### Expression profiling sequencing

Seven days of tomato seedlings were transplanted to the 0.5 × MS medium with 10 μM rapamycin, 10 μM KU63794, and 0.1% DMSO as control and incubated for 24 h, the whole plant including shoot and root were harvested and ground in liquid nitrogen. Three independent biological replicates for each treatment (DMSO, rapamycin or KU63794) were produced. Three individual plants were chosen randomly to prepare one pooled RNA sample as one biological repetition. The RNA extraction, quality evaluation, and cDNA library construction were done as described in previous report (Dong et al., 2015). The libraries were then sequenced using the Illumina HiSeqTM 2000. Genes were defined as differentially expressed if they exhibited a two-fold or greater change between the treatment and control with a false discovery rate (FDR) of 5% or less. All the differentially expressed genes (DEGs) were annotated on the NCBI NR database (http://blast.ncbi.nlm.nih.gov/Blast.cgi). The resulting InterPro and BLAST annotations were performed into GO annotations and all the GO terms were mapped to the GO categories. The significantly functional GO enrichment was evaluated using the Fisher's exact test within Blast2GO (false discovery rate FDR <0.05; Conesa et al., 2005). Significantly enriched KEGG pathways were identified using a hypergeometric test and Benjamini–Hochberg FDR correction and performed using KOBAS2.0 software (http://kobas.cbi.pku.edu.cn/; Xie et al., 2011; Kanehisa et al., 2015). Comparisons of the DEGs got from rapamycin vs. DMSO (rapamycin) and KU63794 vs. DMSO (KU63794) were conducted based on the online Venny analysis (http://bioinfogp.cnb.csic.es/tools/venny_old/index.html; Oliveros, 2007). The heat map was generated from RNA-seq data with software of HemI (Heatmap Illustrator, version 1.0).

### RNA isolation and real-time quantitative RT-PCR (qRT-PCR)

Total RNA of plants was extracted for qRT-PCR and Illumina RNA-seq by using the RNAprep Pure Plant Kit (TianGen Biotech). Total RNA (1 μg) was reverse transcribed by using the PrimeScript RT Reagent Kit (TAKARA). Gene expression was analyzed by quantitative RT-PCR using SYBR® Premix Ex Taq™II (TAKARA) on a Bio-Rad CFX96 Real-time PCR system (Bio-Rad). The primers of the candidate genes and the reference gene *Actin* (Solyc03g078400) are listed in Table S1. Expression was calculated with the comparative cycle threshold (Ct) method, which involves normalizing against the geometric mean of the housekeeping genes (*Actin*) for each treatment.

### Cloning and sequencing of tomato *FKBP12* and *TOR*

The coding sequences of tomato *FKBP12* were amplified by PCR with the primer pairs (Forward: 5′-GCGGCCGCATGGGAGTGGAGAAGGAAGT-3′, Reverse: 5′-CCTGCAGGTTGTGCACCTAGGACTTCAA-3′, N*ot*I and S*bf* I sites underlined). PCR products were cloned into the T1-Simple cloning vector (TransGen Biotech) and sequenced. The full length CDS of *TOR*-like in tomato (XM_004230627.1) was amplified using PrimeSTAR® Max DNA Polymerase Kit (TAKARA) with the primer pairs (Forward: 5′-AACTAGTGCGGCCGCATGGCTGCCACCGTTCAGGCGA-3′, Reverse: 5′-CCCGGGACCTGCAGGCCAAAATGGACACCACCCAAC-3′) which contained 15 bp extensions homologous to entry vector p8GWN ends. And then, the purified PCR product was cloned on p8GWN (linearized by N*ot*I and S*bf* I) by Seamless cloning using In-Fusion® HD Cloning Kit (Clontech) following the user manual. The CDS sequence was verified by sequencing. Sequence analysis was conducted by using programs deposited in the NCBI network (https://www.ncbi.nlm.nih/gov). The deduced amino acid sequences were aligned using the CLUSTAL W 1.81 and the phylogenetic tree was constructed with the MEGA software.

### *Arabidopsis thaliana* growth and transformation

*Arabidopsis thaliana* L. (Columbia ecotype) was used in this study. To generate gateway entry vector constructs, the *SlFKBP12* fragment was inserted into the downstream of CaMV 35S promoter and fused HA-tag sequence carrying p8GWN Entry vector via digestion with N*ot*I/S*bf* I, the experimental protocols were described in previous report (Ren et al., 2011). Then the expression cassette was introduced pEarleyGate303 (pEG303) by using the LR Clonase II reaction (Life Technologies). The pEG303 is the destination vector of a Gateway system established by previous report (Earley et al., 2006). The resulting destination vectors were used for plant transformation. The floral dipping method was employed for generating transgenic plants (Zhang et al., 2006). The transformation and screening of primary transformants were performed according to Zhang's method (Zhang et al., 2006).

### Inhibitory effect measurements of rapamycin on *SlFKBP12*-expressing lines

*SlFKBP12*-expressing lines of T3 generation were grown on the 0.5 × MS plates with differing concentrations of rapamycin (0, 0.5, 1, 5, and 10 μM) at 22°C and 16-h/8-h light/dark cycle. The fresh weight and root length were measured as described in previous report (Xiong et al., 2016). The hypocotyl lengths of *SlFKBP12*-expressing lines were measured after plants were grown on 0.5 × MS medium supplemented with 10 μM rapamycin in dark for 5 days. *ScFKBP12*-expressing plants were previously generated by Ren et al. were set as rapamycin sensitive controls (Ren et al., 2012). Root hair was photographed by using the OLYMPUS MVX10 stereoscopic microscopes (Olympus, Japan).

### Protein extraction and immunoblotting

To extract the protein, 100 mg fresh plant sample were collected from wild type *Arabidopsis, ScFKBP12,*- and *SlFKBP12*-expressing lines, respectively, then ground in liquid nitrogen and quickly transferred into a 1.5 mL EP tube for further protein extraction. Four hundred microliters of RIPA lysis buffer (including 10 mM Tris, pH 8, 150 mM NaCl, 2% Triton, 1 mM PMSF, protease inhibitor, and 10 mM CaCl2, Sigma) were added to the above EP tubes and incubated together for 1 h at 4°C with constant shaking, and then centrifuged for 5 min at 12,000 rpm at 4°C to remove cell debris. Subsequently, 200 μL supernatant mixed with 40 μL 6 × protein loading buffer were added to the EP tubes, and boiled in boiling water for 5 min. After incubation, the samples were separated by SDS-PAGE, and the SlFKBP12 protein was detected by immunoblotting with antibodies (EarthOx) against HA-Tag. For the SlTOR detection, extracted protein from tomato seedlings was detected by immunoblotting using polyclonal antibody against Arabidopsis TOR(N) (Abiocode) based on high amino acid sequence identity between SlTOR with AtTOR. ECL Western Blotting Detection Reagents (Thermo) were used for detection.

For the protein gel shifting assay, 5DAG seedlings of *ScFKBP12*- or *SlFKBP12*-expressing *Arabidopsis* line were collected after treated with various concentration rapamycin (0, 1, 5, and 10 μM) for 24 h, and then divided into two groups. Then the total proteins were extracted by using the denaturing lysis buffer and native lysis buffer according to the protocols of Minute™Total Protein Extraction Kit for Plant Tissus (Invent Biotech). The extracted proteins after treated with denaturing lysis buffer were mixed with 6 × protein loading buffer and boiled for denaturation, then the proteins were separated in 10% SDS-PAGE and detected by immunoblotting. Another group of extracted proteins with native lysis buffer were mixed with 4 × native protein loading buffer before separated in 10% SDS-PAGE.

### TOR inhibitors combination assays

For TOR inhibitors combination assays, the surface-sterilized tomato seeds were treated with 10 μM rapamycin, 10 μM KU63794, or the two drugs combination. After 7 days of incubation, the fresh weight of seedlings was measured for evaluating inhibitory effects. As for transgenic *Arabidopsis*, sterilized seeds from *SlFKBP12*-expressing line 8, *ScFKBP12*-expressing line, and wild type were incubated on 0.5 × MS plate supplemented with 5 μM rapamycin, 5 μM KU63794, or their combination. Ten days after germinatioin, the seedlings were photographed and the fresh weight of seedlings was measured to assess the growth inhibition.

## Results

### Molecular components of TOR complex in tomato

**Figure 1:**
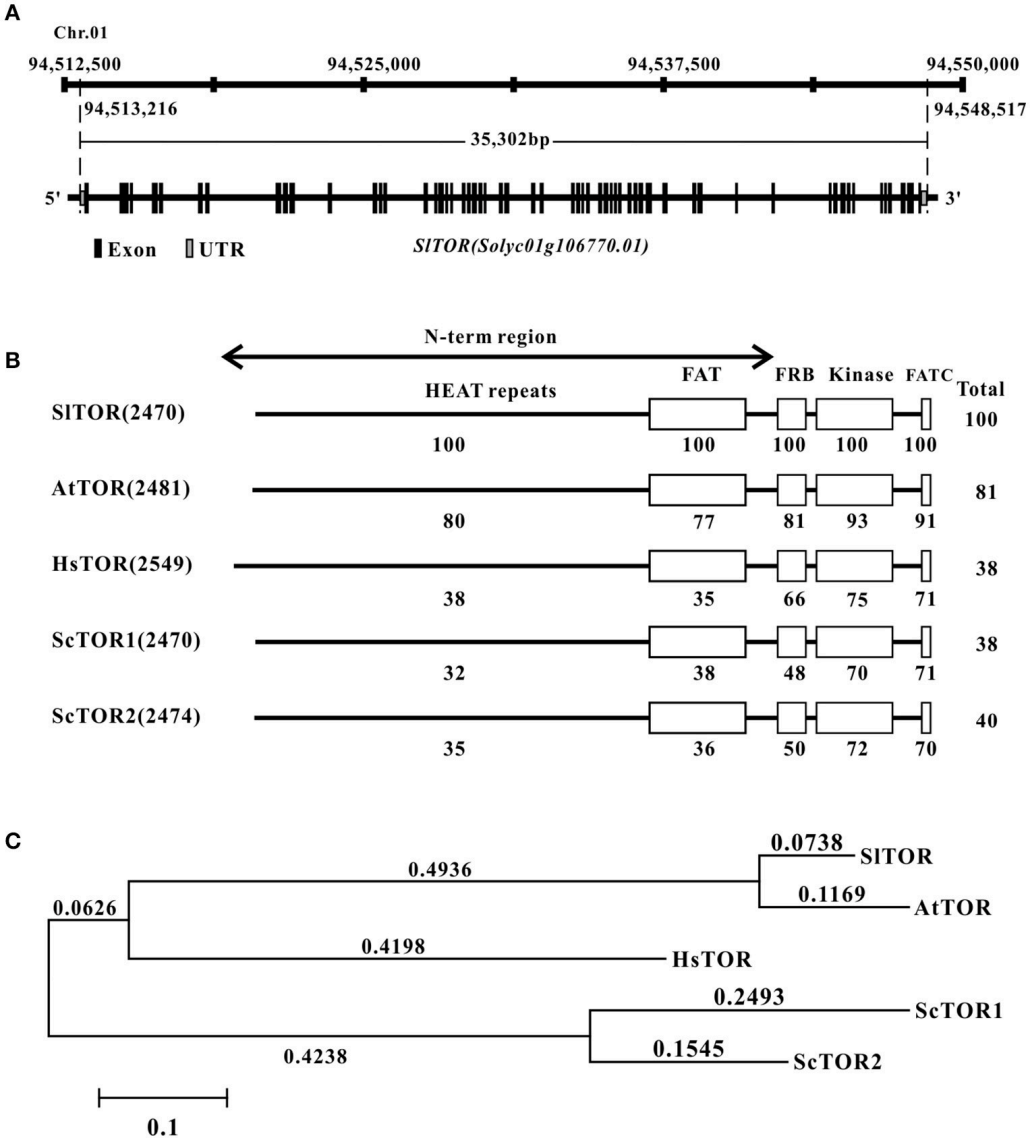
**The information of ***TOR*** homolog in tomato. (A)** The gene locus and structure of *TOR homolog* in tomato. **(B)** Domain organization of SlTOR protein and comparison of the SlTOR amino acids sequences with that of TOR proteins from other organisms. **(C)** Phylogenetic analysis of tomato TOR with that from other species. *Sl, Solanum lycopersicum; At, Arabidopsis thaliana; Hs, Homo sapiens; Sc, Saccharomyces cerevisiae*. HEAT repeats: Huntingtin, Elongation factor 3, A subunit of protein phosphatase 2A, and TOR1; FAT: FRAP, ATM, and TRRAP domain; FRB: FKP12-rapamycin binding domain; FATC: Carboxy-terminal FAT domain.

Tomato *TOR* gene (*SlTOR*) was found through questing for the homologs of *Arabidopsis TOR* coding region sequence (CDS) against tomato genome (Tomato Genome, 2012; https://solgenomics.net/) and NCBI (http://www.ncbi.nlm.nih.gov/), respectively. The results shows that a single *TOR* homolog gene (Solyc01g106770) locates on chromosome 1 of tomato (Figure 1A). However, 18 bases pair nucleotide acids of *TOR*-like are missing in the version of Sol Genomics Network compared with that of NCBI database (GenBank accession no. XM_010316867). To confirm the sequence of CDS of *TOR* in Micro TOM, the full length CDS of *TOR* has been amplified and cloned (Figure S1A). The sequencing results show that the CDS of *TOR* is identical to the version of NCBI rather than Sol Genomics Network. Based on this sequence, we find that full length genome of *SlTOR* spans about 35.3 kb and contains 57 exons and 56 introns (Figure 1A).

The tomato TOR protein sequence seems to consist of 2470 amino acid residues with a predicted molecular mass of 278 kDa and the western blotting result showed that the protein size of SlTOR is similar to that as of AtTOR (Figure S1B). Alignment of SlTOR with TOR protein sequences from other organisms shows consistent domain organization with a high identification and conservation of the FRB and kinase domains as well as in a C-terminal domain named FATC (Figure 1B), which is conserved in the phosphatidylinositol 3-kinase related protein kinase (Schmelzle and Hall, 2000; Fruman and Rommel, 2014). Highly conserved amino acids are also present throughout the N-term region (Figure 1B), which contains repeat motif sequence named HEAT repeats and have been reported to be in relation to proteins interactions (Schmelzle and Hall, 2000; Hara et al., 2002; Xiong and Sheen, 2014). Phylogenetic analysis shows a closer evolutionary relationship between SlTOR and AtTOR than with the other TORs (Figure 1C).

In yeast or animals, TOR protein participates in forming two structurally and functionally distinct multiprotein complexes, TORC1 and TORC2, and each complex recruits shared and distinct TOR interacting components (Helliwell et al., 1994; Xiong and Sheen, 2014). As *Arabidopsis*, in tomato, two genes encoding RAPTOR homologous on chromosomes 9 (Solyc09g014780) and 10 (Solyc10g076260), but one gene encoding LST8 (Solyc03g059310) homolog was found, which are the core components of TORC1. By contrast, there is no evidence of a plant TORC2 complex to date due to specific components of this complex, RICTOR, was not found in tomato (Table 1). The results indicate the existence of a conserved and functional TORC1 in tomato.

**Table 1:** **TORC1, TORC2 homologs in tomato as compared to various species**.

	***Hs***	***Sc***	***At***	***Sl***
TORC1	mTOR	TOR1/2	TOR	TOR
	RAPTOR	Kog1	RAPTOR1A/1B	RAPTOR1A/1B
	LST8	Lst8	LST8-1/2	LST8
	–	Toc89	–	–
	PRAS40	–	–	–
	DEPTOR	–	–	–
TORC2	mTOR	TOR2	TOR	TOR
	SIN1	Avo1	–	–
	–	Avo2	–	–
	RICTOR	Avo3	–	–
	LST8	Lst8	LST8-1/2	LST8
	PRR5	Bit61	–	–
	DEPTOR	–	–	–

*At, Arabidopsis thaliana; Hs, Homo sapiens; Sc, Saccharomyces cerevisiae; Sl, Solanum lycopersicum. TORC1, target of rapamycin complex 1; TORC2, target of rapamycin complex 2; mTOR, mammalian target of rapamycin; DEPTOR, DEP domain-containing mTOR-interacting protein; RAPTOR, regulatory-associated protein of mTOR; RHEB, RAS homolog enriched in brain; RICTOR, rapamycin-insensitive companion of mTOR; TSC1/2, tuberous sclerosis complex 1/2. (–) indicates that there are no shown/obvious homologs of the indicated proteins in the corresponding organisms*.

### Growth inhibition by both rapamycin and active-site TOR inhibitors (asTORis) in tomato

**Figure 2:**
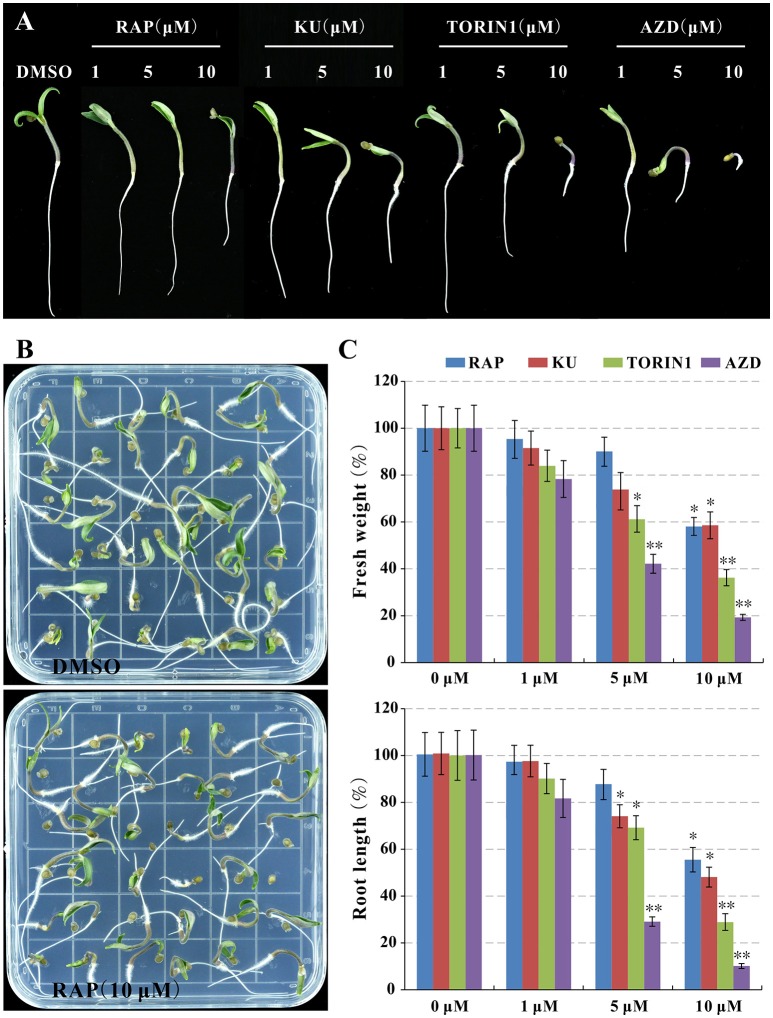
**Rapamycin and asTORis display inhibitory effects on tomato seedlings growth. (A,B)** The phenotype of seedlings after 7 days of germination. **(C)** The fresh weight and primary root length in **(B)** were measured. Values represent mean ± SEM. Significant differences from the control are indicated by ^*^P < 0.05, ^**^P < 0.01. Each concentration contains three biological replicate.

In order to understand TOR signaling in tomato, asTORis (KU63794, TORIN1, and AZD8055) was applied to treat tomato seedlings, meanwhile, rapamycin also was used to test rapamycin-sensitivity of tomato. It is surprising that the tomato seedling growth was observed obvious growth inhibition reflecting on reducing fresh weight and root length (Figures 2A,B) at 10 μM rapamycin compared to the control indicated that SlFKBP12 may be functional in bridging the interaction between rapamycin and tomato TOR (Figure 2). Similar to wild type *Arabidopsis* treated with different asTORis (Montané and Menand, 2013; Dong et al., 2015), the dosage-dependent effect of asTORis on fresh weight and primary root length were observed when tomato was treated with the asTORis (KU63794, TORIN1, and AZD8055; Figure 2). In addition, the 5 DAG (days after germination) tomato seedling were transferred to 0.5 × MS medium supplemented with different inhibitors (rapamycin, KU63794, TORIN1, and AZD8055) to further assess inhibitory effects on seedlings growth. After 5 days growing with the inhibitors, the tomato seedling growth was inhibited in all the inhibitors tested (Figure S2). Taken together, these results imply that a functional FKBP12 existing in tomato and a conserved SlTOR function on controlling the growth of tomato seedlings.

### Identification and cloning of *SlFKBP12*

**Figure 3:**
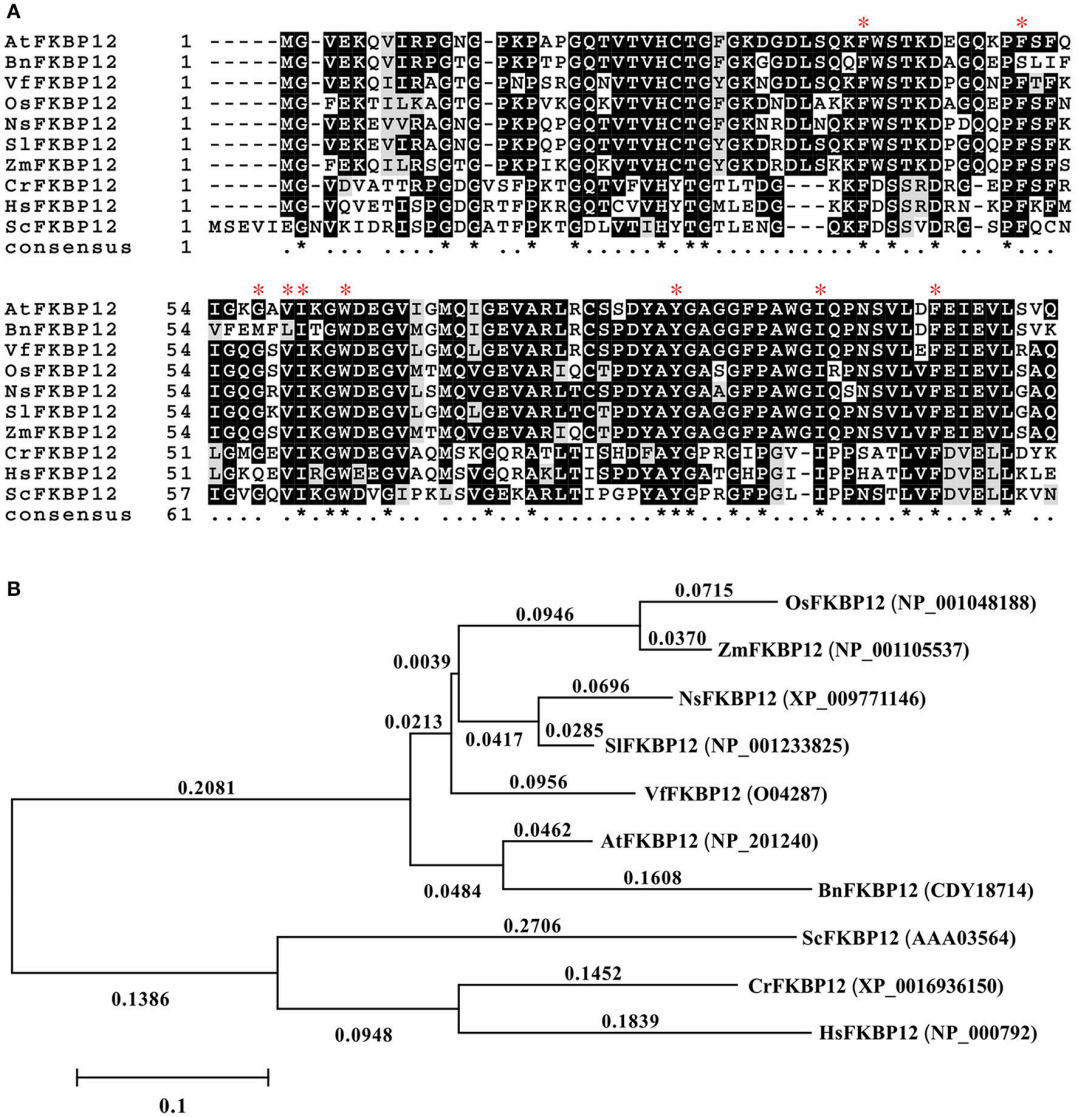
**Comparison of the FKBP12s among different organisms. (A)** Multiple alignments based on the FKBP12 sequences from various species. The red asterisk indicates the key site of HsFKBP12 for interacting with rapamycin corresponding to that in quarternary structure. **(B)** Phylogenetic analysis of the SlFKBP12 and representative organisms. The numbers means the rate of substitution of amino acid. Cut-off value for consensus tree is 50%. The scale bar represents the estimated number of amino acid substitutions per site. *Sl, Solanum lycopersicum; At, Arabidopsis thaliana; Bn, Brassica napus; Ns, Nicotiana sylvestris; Vf, Vicia faba; Zm, Zea mays; Os, Oryza sativa; Cr, Chlamydomonas reinhardtii; Hs, Homo sapiens; Sc, Saccharomyces cerevisiae*.

The rapamycin response in eukaryotes depends on the formation of the ternary complex of rapamycin-FKBP12-FRB domain of TOR. The conservation of SlTOR with other TORs and the interchangeable FKBP12 in plants drove us to mine information on *SlFKBP12* to understand the growth inhibition of tomato caused by rapamycin (Xu et al., 1998; Menand et al., 2002; Crespo et al., 2005; Agredano-Moreno et al., 2007). The amino acid sequence of SlFKBP12 (Solyc01g105710, GenBank accession no. NP_001233825) was downloaded from the Sol genomics network (https://solgenomics.net/) and aligned to various FKBP12s from other eukaryotic organisms (Figure 3A). The identity between the SlFKBP12 protein and its homologs in other organisms ranges from 47 to 90% at the amino acid level. Phylogenetic development analysis showed *S. lycopersicum* had the closest relationship with *Nicotiana sylvestris* (Figure 3B). The drug-binding pocket of HsFKBP12 formed by several hydrophobic residues (Phe36, Phe46, Val55, Ile56, Trp59, Tyr82, Ile91, and Phe99) in quarternary structure is conserved in the SlFKBP12 sequence (Van Duyne et al., 1993). The glycine (Gly53) residues, which are involved in the formation of X-sheet, and loop-loop packing (Van Duyne et al., 1991), are also conserved (Figure 3A). These conserved features also suggest that SlFKBP12 proteins maintain a secondary structure with a five-stranded X-sheet, a 40's loop, and an 80's loop that are characteristic of the human FKBP12 (Clardy, 1995).

### Expression of *SlFKBP12* in *Arabidopsis* confers sensitivity to rapamycin

**Figure 4:**
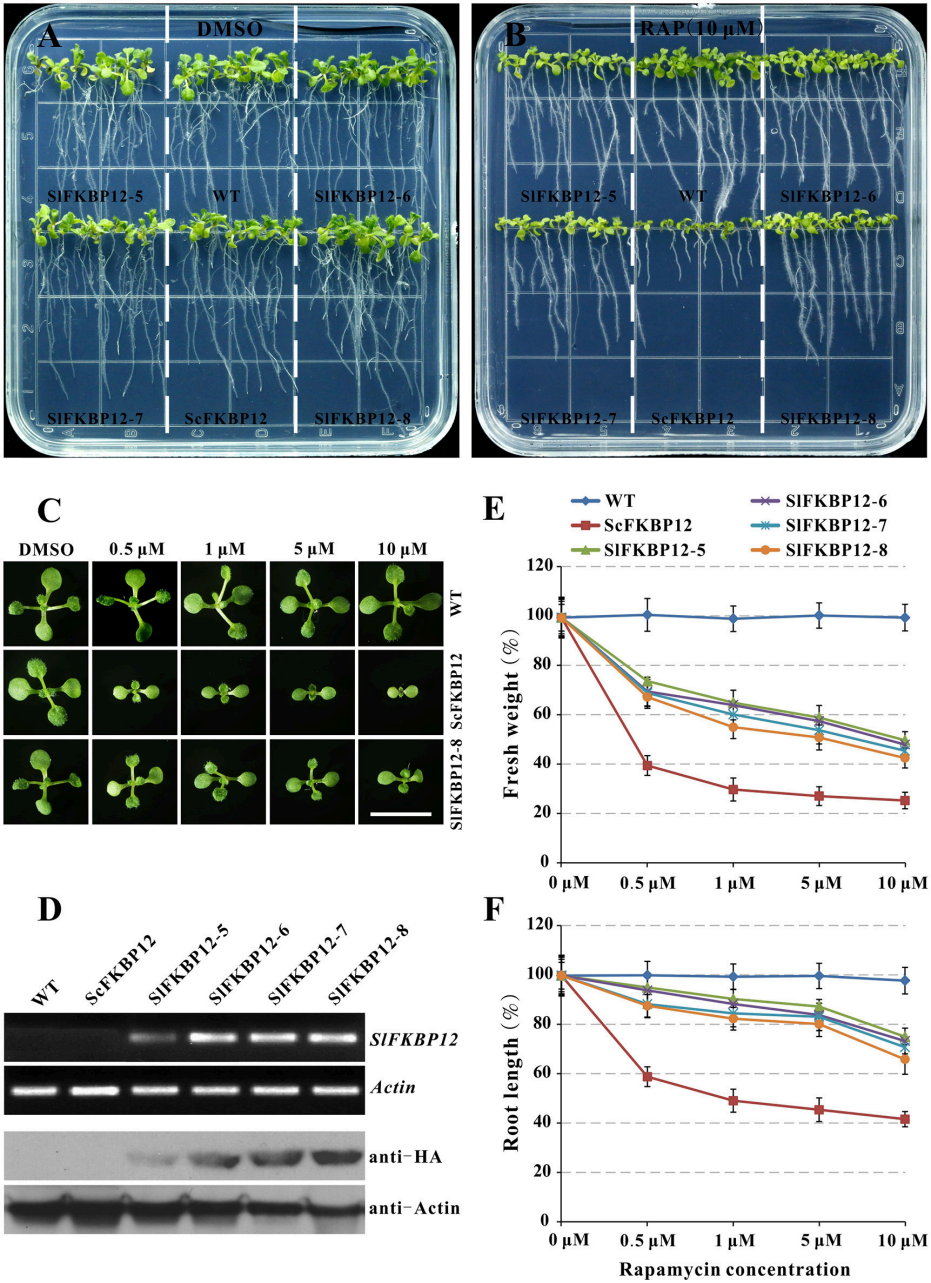
**SlFKBP12 restores rapamycin sensitivity in Arabidopsis. (A,B)** 10DAG seedlings under the treatment of DMSO and 10 μM rapamycin, respectively. **(C)** Cotyledons of WT, ScFKBP12, and SlFKBP12 T2-8 at 10DAG with rapamycin at different concentrations. Bar = 1 cm. **(D)** The detection of SlFKBP12 in transcript and protein levels. **(E,F)** Dosage-dependent curves of rapamycin for plant fresh weight and primary root length for above different *Arabidopsis* lines were measured at 10 DAG, respectively. DAG, day after germination.

Early studies showed that the FKBP12 protein from yeast or humans could restore the rapamycin sensitivity in *Arabidopsis* (Mahfouz et al., 2006; Sormani et al., 2007; Ren et al., 2012). To further confirm the SlFKBP12 function on bridging the interaction between TOR and rapamycin, the coding region of *SlFKBP12* was cloned and introduced into *Arabidopsis*. As shown in Figure 4A, no obvious morphological phenotypes appeared in any of these transgenic lines. However, the transgenic lines with *SlFKBP12* displayed response to rapamycin as *Arabidopsis* expressing *ScFKBP12* (Ren et al., 2012) on a solid cultured conditions. Results showed that the inhibitory effect of rapamycin on primary root length and plant fresh weight was most effective in the *ScFKBP12* line, followed by *SlFKBP12*-8, 7, 5, and 6 (Figures 4A–F). At concentrations around 0.5 μM rapamycin, all the transgenic lines displayed smaller cotyledon and leaves reflecting in the decreasing fresh weight compared with WT (Figure 4C). With increasing concentrations of rapamycin, the transgenic lines were increasingly inhibited. In the dark, rapamycin could repress the hypocotyl elongatioin of all the *SlFKBP12*-expressing *Arabidopsis* lines significantly, similar to the *ScFKBP12*-expressing line, while there was no obvious difference observed between the treatment of rapamycin and DMSO in WT (Figure S3A). At 5 days after germination, hypocotyl elongation inhibition was about 50% in all the transgenic lines comparing rapamycin to DMSO (Figure S3B). These results suggest that *SlFKBP12* can partly restore sensitivity to rapamycin in *Arabidopsis*.

### SlFKBP12 can mediate the interaction between rapamycin and TOR

**Figure 5:**
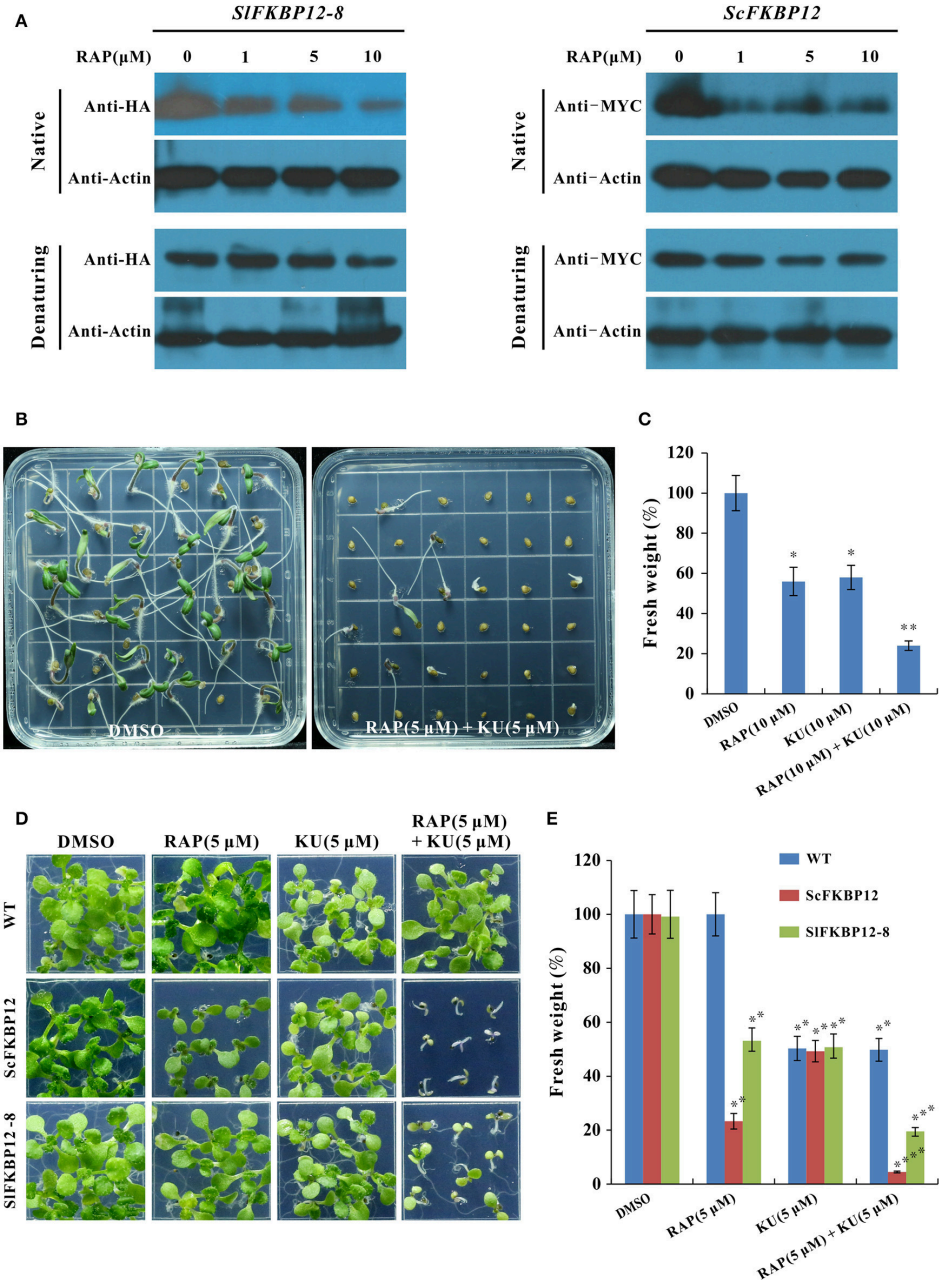
**SlFKBP12 mediates the interaction between rapamycin and TOR. (A)** The SlFKBP12 and ScFKBP12 level detected by western blotting in native and denaturing protein after treated with rapamycin (Please see Figure S5 for the original images of gel shifting assay). **(B–E)** The combination of rapamycin and KU63794 significantly enhances the growth inhibition. **(B)** Representative images of tomato seedling phenotype after germination on MS medium supplemented with rapamycin combined with KU63794 and dimethyl sulfoxide (DMSO) (0.1%) was set as control. **(C)** The fresh weight of tomato seedlings. **(D)** Representative images of WT, ScFKBP12, and SlFKBP12-expressing line of 10 DAG incubated on 0.5 × MS with rapamycin, KU63794 and the two drugs combination. **(E)** The fresh weight of the *Arabidopsis* seedling in **(D)** were measured. Error bars indicate ± SD for triplicates. Asterisks indicate significant differences using Student's *t*-test compared with the DMSO treatment (^*^P < 0.05, ^**^P < 0.01, ^***^P < 0.001; ^****^P < 0.0001).

To further confirm that SlFKBP12 are able to brigde the interaction between rapamycin and TOR. Protein gel shifting assay was performed to detect the band difference between the free monomer FKBP12 protein and FKBP-RAP-TOR complex in *ScFKBP12* and *SlFKBP12*-expressing plants (SlFKBP12-8) after treated with various concentration of rapamycin (0, 1, 5, and 10 μM). As shown in Figure 5A, with increasing of rapamycin, SlFKBP12 protein level displayed a decline trend in native stage but not denaturing molecular stage (Figure 5A), indicating that there was an increasing FKBP-RAP-TOR complex formation with the increasing rapamycin concentrations. Similar results also were observed in *ScFKBP12-expressing* plants (Figure 5A). These results suggest that, like ScFKBP12, SlFKBP12 has the functions to bridge the interaction between TOR and RAP in plants. However, the FKBP-RAP-TOR complex was not detected in the gel shifting assay. The possible reason is that the molecular weights of TOR, RAPTOR, and LST8 protein are 280, 150, 35 KD, respectively. The total molecular weight of TOR complex (containing TOR, RAPTOR, LST8, FKBP12, rapamycin, and some unknown proteins) is more than 500 KD. Usually, it is very difficult to transfer a big protein from gel onto membrane if the protein molecular weight is more than 250 KD.

It was reported that rapamycin and asTORis combination could create significant addictive or synergistic inhibitory effects on rapamycin sensitive *Arabidopsis* plants due to rapamycin and asTORis binding to various target sites of TOR (Deng et al., 2016; Xiong et al., 2016). We therefore examine the effect of rapamycin combined with asTORis on tomato and *SlFKBP12*-expressing *Arabidopsis*. As shown in Figure 5B, comparing to rapamycin and KU63794 alone treatment (Figure 2), the combination of these two drugs showed more severe inhibitory effect on tomato seedlings growth after 7 days incubation. The fresh weight reduced to about 20% when treated with rapamycin and KU63794 combination compared with single drug treatment (Figure 5C). These results indicate that the additive or synergistic effects can be created by rapamycin and KU63794 combination in tomato. Similar results also were observed on *SlFKBP12*-expressing *Arabidopsis* when treated with rapamycin and KU63794 combination (Figure 5D) and the additive or synergistic inhibitory effects is identical to that *ScFKBP12* plants after same treatment (Figures 5D,E). Taken together, our data indicate that SlFKBP12 could bridge the interaction between rapamycin and TOR.

### Comparative analysis of gene expression profile between rapamycin and KU63794 treatment of tomato

**Figure 6:**
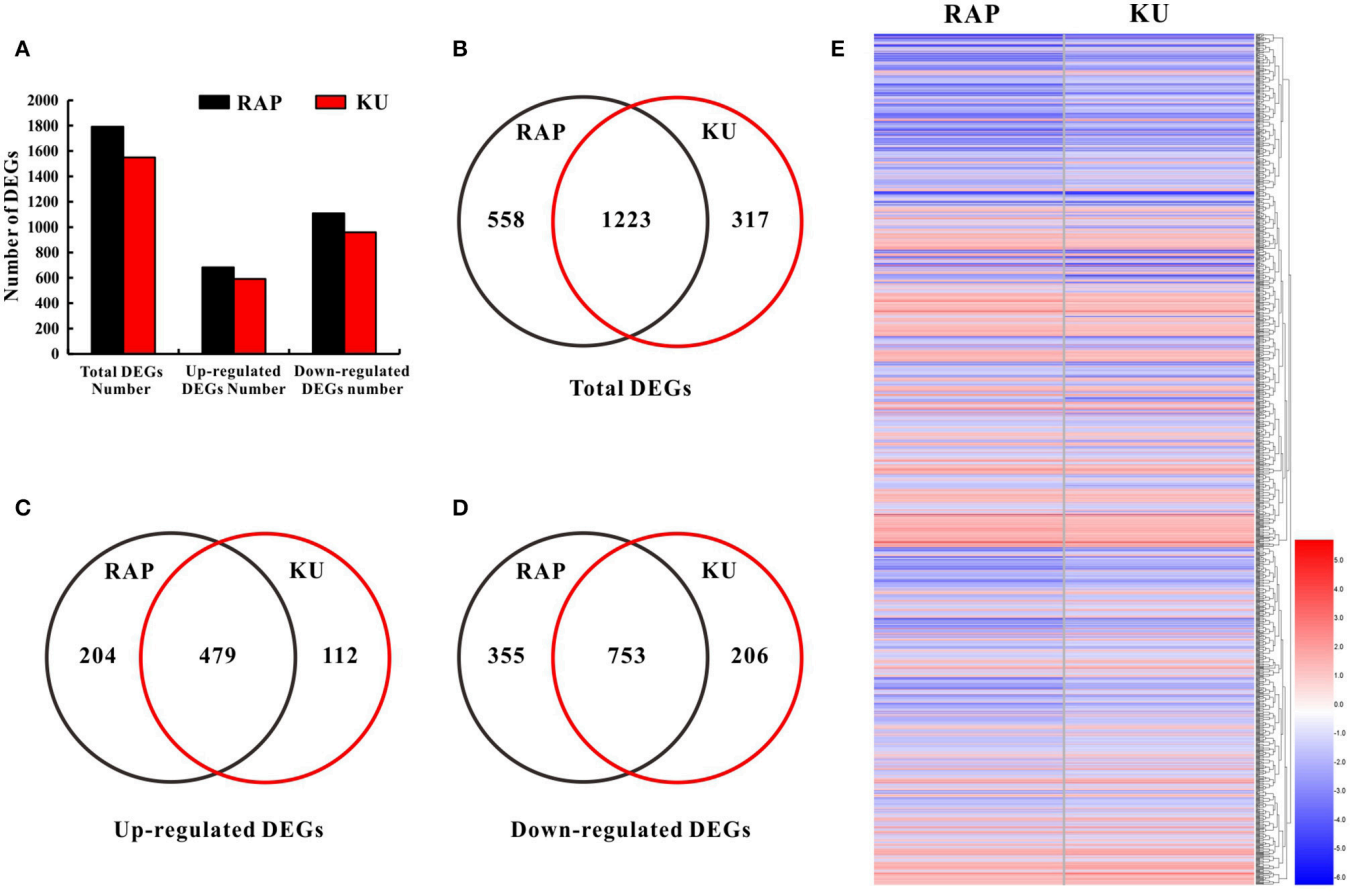
**Comparative analysis of DEGs under the treatment of rapamycin and KU63794. (A)** The number of DEGs from the RNA-Seq data. **(B)** The number of total DEGs shared between rapamycin and KU63794 treatment is represented by overlapping circles. **(C,D)** The number of up-regulated and down-regulated genes shared between rapamycin and KU63794 treatment is represented by overlapping circles, respectively. **(E)** DEGs in the comparison between rapamycin and KU63794 treatment. RAP, rapamycin; KU, KU63794.

To further seek the function of SlFKBP12 and TOR signaling on tomato seedling growth, whole gene expression profile analysis of tomato seedling growth under TOR inhibition was performed by RNA-Seq. Rapmycin and KU63794 are very specific TOR inhibitors and lack non-specific targets to other kinases in plants (Tables S2, S3). Therefore, rapamycin and KU63794 were selected to treat seedlings for investigating the early molecular landscape in response to TOR suppression. RNA Sequencing for global expression profiling changes were conducted in tomato after the treatment of 24 h 10 μM rapamycin, 10μM KU63794, and corresponding volume of DMSO as control, respectively. As shown in Figure 6B, 1791 DEG's (1108 down-regulated and 683 up-regulated) out of 34,191 were identified in rapamycin treatments (Tables S4A,C), and 1550 DEGs (959 down-regulated and 591 up-regulated) out of 34,723 genes identified from KU63794 (Tables S4B,D). All the DEGs were annotated in the NCBI NR database (http://blast.ncbi.nlm.nih.gov/Blast.cgi) and the Sol Genomics Network website (https://solgenomics.net/; Tables S4C,D). The two treatments shared 1233 overlapping DEGs and nearly all of the overlapping genes (753 down-regulated and 479 up-regulated) showed the same tendency of up-regulated or down-regulated expression (Figures 6C–E, Table S4E). Some overlapping DEGs were selected randomly from the RNA-Seq data and the same trends were validated by real time-PCR (Figure S4). There were 5 genes in the top 15 down-regulated genes in both the treatment of rapamycin and KU63794, while 7 genes among the top 15 up-regulated genes, were observed simultaneously in both treatments (Table S5). Our data reflects that rapamycin and KU63794 likely have overlap on function as TOR inhibitors.

**Figure 7:**
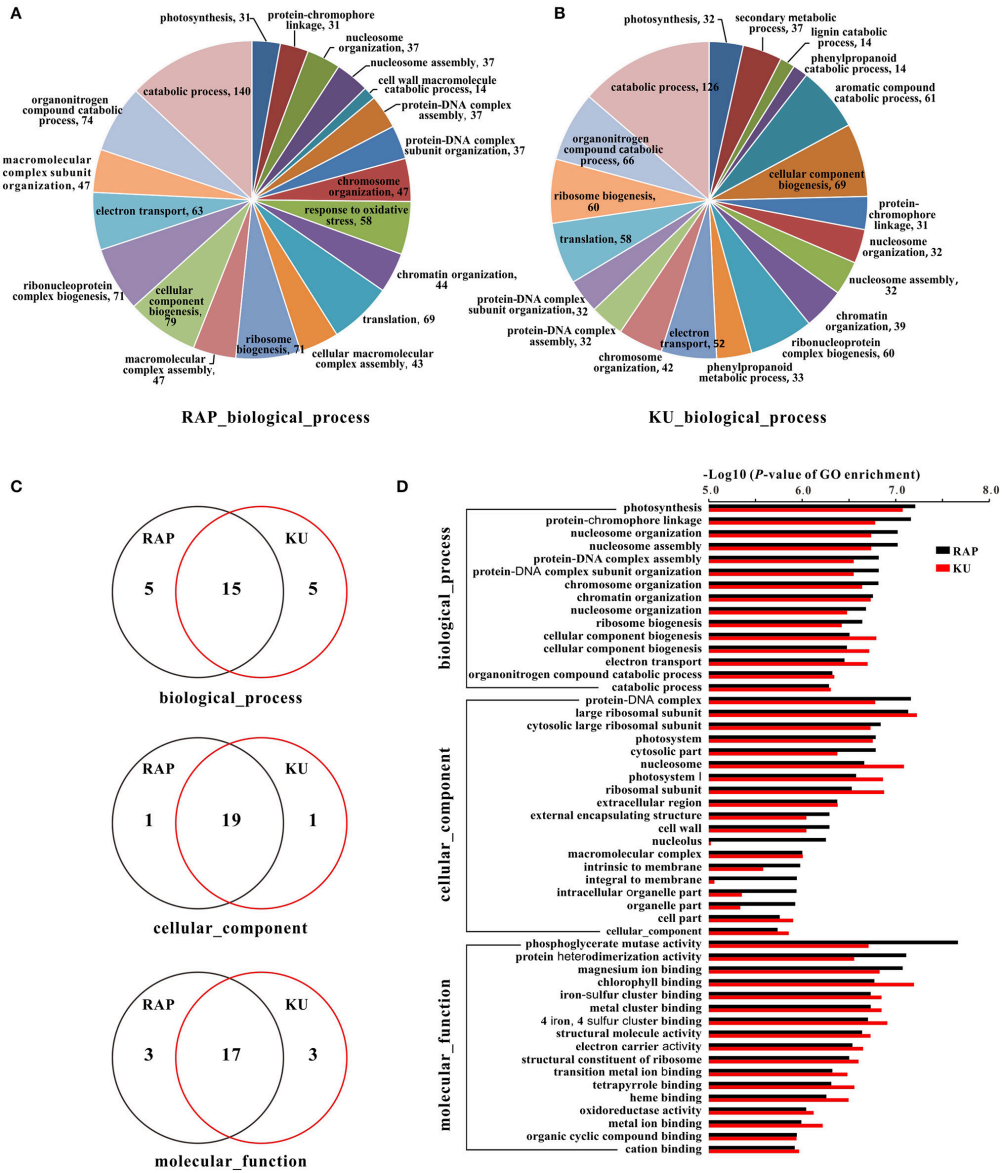
**The GO information of tomato seedlings when treated with rapamycin and KU63794. (A,B)** The top 20 GO terms in biological process under the treatment of rapamycin and KU63794, respectively. **(C)** The GO terms shared between rapamycin and KU63794 treatment are represented by overlapping circles. **(D)** GO assignments under the treatment of rapamycin and KU in tomato. RAP, rapamycin; KU, KU63794.

To further understand the function of these DEGs, GO assignments, KEGG pathways, and their enrichments were analyzed, respectively. Approximately 70.18% DEGs from the rapamycin data and 70.32% DEGs from the KU63794 data were assigned to one or more of three GO categories: cellular component (CC), molecular function (MF), and biological process (BP). A total of 282 and 281 GO terms were enriched in tomato under the TOR inhibition with rapamycin and KU63794, respectively (Tables S6A,B). Of the GO category biological process, the top 20 enriched GO terms are showed on Figures 7A,B. With respects to Gene Ontology, there were 15, 19, and 17 GO terms overlapped in the BP, CC, and MF categories, respectively, for both treatments (Figures 7C,D). In addition, 19 out of 183 KEGG pathways were enriched in the rapamycin data, and 18 out of 184 KEGG pathways were enriched in the KU63794 data (Tables S7A,B). High overlapping of enrichments in GO assignments and KEGG pathways suggest that rapamycin and KU63794 share a similar molecular mechanism in repressing TOR activity in tomato.

**Figure 8:**
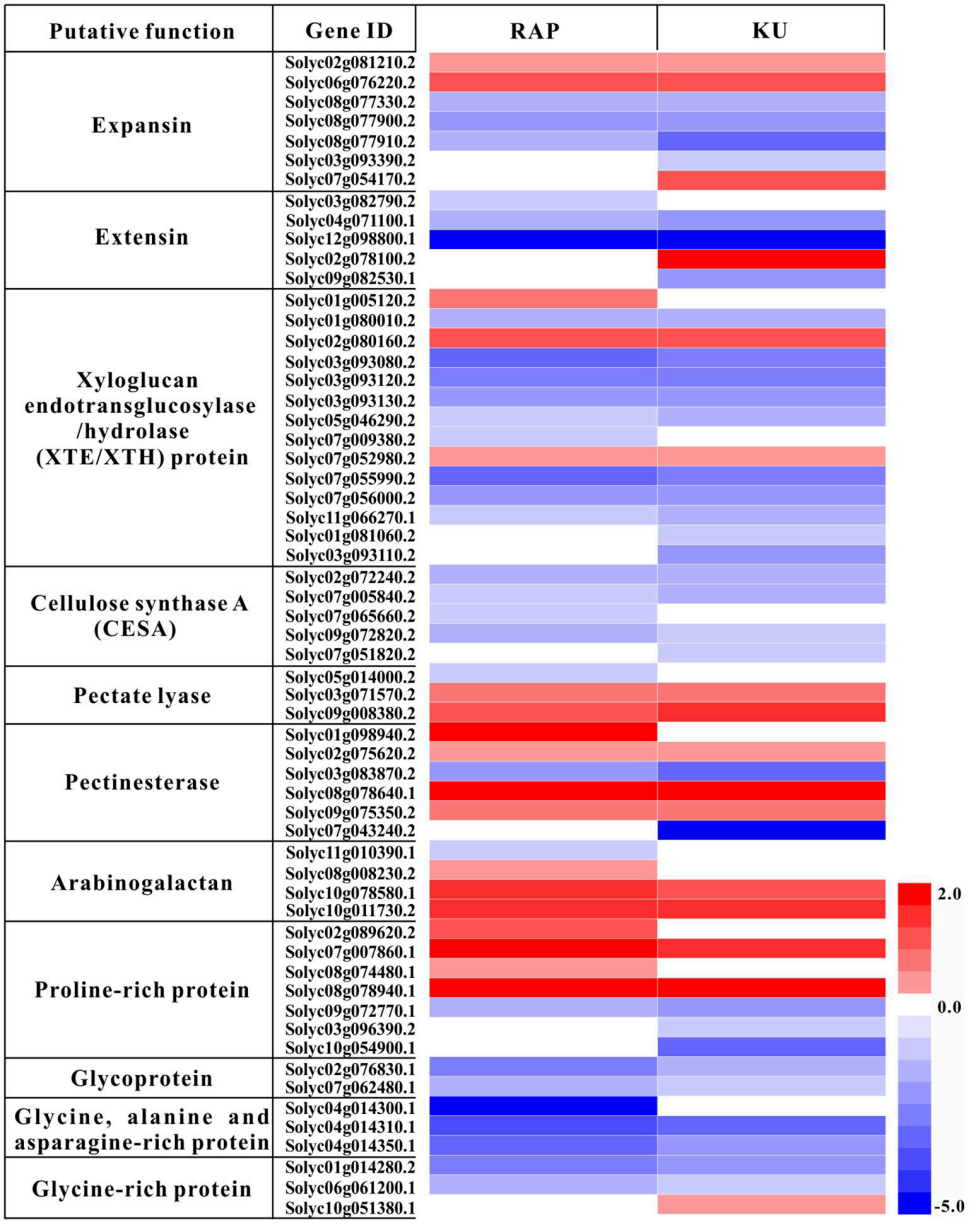
**Heat map analysis of the rapamycin and KU63794 showed the differential expression gene involved in cell wall expansin**. Red text indicates significantly up-regulated genes. Blue text indicates genes that were significantly down-regulated. RAP, rapamycin; KU, KU63794.

The overlapping enriched GOs and KEGG pathways include many TOR conserved functions identified in *Arabidopsis*, such as cell wall biogenesis, photosynthesis, transporters. In this study, total of 59 DEGs involved in photosynthesis were detected under the treatment with either rapamycin or KU63794 (Table S8). The “photosynthesis (light reaction)” KEGG pathway was one of the most enriched pathways in both treatments (Table S8). Out of 47 DEGs, there were 34 down-regulated, overlapping genes, indicating that light reaction associated genes were significantly repressed under the inhibition of TOR with rapamycin and KU63794. In the category of cell wall biogenesis, down-regulated genes included extensins, expansins, xyloglucan endotransglycosylase/hydrolase (XTE/XTH), and cellulose synthase (CESA), etc. (Figure 8, Table S9). For example, there were eight overlapping down-regulated genes encoding XTE. In cell wall restructuring, the expansins and extensins function in loosening the cell wall to slide the microfibril matrix network. The enzyme XTE/XTH is used for cutting the xyloglucans (XG). CESA is employed to connect monosaccharides to the end of polysaccharides to synthesize the cellulose chains. Transporters that are essential for anabolic processes in plants were also down-regulated both in rapamycin- and KU63794-treated tomato. The transporters included boron transporters, nitrate transporters, sulfate transporters, phosphate transporters, zinc transporters, etc., (Table S10). Taken together, these data indicates that TOR plays a vital role in regulating tomato seedling growth.

## Discussion

TOR is a master growth regulator among yeast, plants, animals and humans (Wullschleger et al., 2006; Xiong and Sheen, 2014). The TOR pathway in yeast and mammals is much clearer than that in the plants, partially attributed to the rapamycin sensitivity in yeast and mammals (Wullschleger et al., 2006; Xiong and Sheen, 2014). The resistance to rapamycin for the most prevailing land plants hampered TOR research in plants (Xu et al., 1998; Menand et al., 2002). In this study, a tomato variety *S. lycopersicum*, cv. Micro Tom was inhibited by rapamycin (Figure 2, Figure S2), similar to *Zea mays* and *Chlamydomonas creinhardtii* (Crespo et al., 2005; Agredano-Moreno et al., 2007). The different response to rapamycin in plants suggests that they might have different regulatory mechanisms for TOR signaling. The discovery of sensitive-to-rapamycin WT plants will provide easy-to-obtain materials for further TOR pathway research, thus facilitating comparisons within the TOR pathway in different plants. Meanwhile, WT tomato is sensitive to rapamycin and thus will facilitate TOR pathway research for fruit development in the future. Basic information on the TOR protein and other core components of TORC1 were identified in tomato by scanning the tomato genome using the bioinformatics methods (Figure 1, Table 1). The high identities of the FRB and kinase domain were obtained among SlTOR and other systems' TORs (Figure 1), suggesting TOR is a structurally conserved protein in a wide range of organisms (Xiong and Sheen, 2014).

Over the past 20 years, rapamycin was extensively used to study the regulatory mechanism of TOR in yeast and mammals (Benjamin et al., 2011). In plants, rapamycin-sensitive transgenic *Arabidopsis* lines expressing *ScFKBP12* and *HsFKBP12*, accelerated the plant TOR research (Mahfouz et al., 2006; Sormani et al., 2007; Ren et al., 2012). Recently developed ATP-competitive kinase domain inhibitors provide a more comprehensive TOR inhibition. In this study, both rapamycin and asTORis were selected to treat tomato seedling for revealing the functions of TOR in tomato. TOR protein is the shared target for rapamycin and asTORis, but rapamycin targets the FRB domain of TOR while asTORis targets the kinase domain of TOR. Rapamycin and asTORis function in parallel to inhibit different domain of TOR. As shown in Figure 5C, the additive or synergistic effects can be created by rapamycin and KU63794 combination in tomato. These data in tomato are highly consistent with the observations in *Arabidopsis* (Xiong et al., 2016), indicating that TOR signaling is conserved in plants, and rapamycin and KU63794 parallelly inhibit TOR activity in the same pathway. As expected, huge overlapping DEGs (1223, Figure 6) detected between rapamycin vs. DMSO and KU63794 vs. DMSO confirm that rapamycin and KU63794 may co-target the TOR pathway. The functional analysis of overlapping DEGs supports a conserved TOR function including, cell wall biogenesis, inorganic nutrition transporters (Tables S8–S10), etc., reflecting conserved TOR function among eukaryotes (De Virgilio and Loewith, 2006; Wullschleger et al., 2006; Benjamin et al., 2011; Xiong and Sheen, 2014). Cell wall biogenesis is unique process in plants compared with other systems. In *Candida albicans*, Rhb1 is involved in the cell wall integrity pathway, by transferring signaling through the TOR kinase and the Mkc1 MAP kinase pathway, thus the TOR signaling pathway participates in establishing cell wall integrity (Tsao et al., 2009). In *Arabidopsis*, inhibition of TOR signaling by rapamycin caused specific changes to galactan/rhamnogalacturonan-1 and arabinogalactan protein components of cell walls (Leiber et al., 2010). Meanwhile, many genes related to cell wall modification, such as extensins, expansins, Pro-rich proteins, and arabinogalactan- and Hyp-rich glycoproteins, were activated after long-term TOR repression using multiple methods (Moreau et al., 2012; Ren et al., 2012; Caldana et al., 2013; Dong et al., 2015). Similar to our results, about 69 genes encoding the cell wall structural proteins, including expansins, extensins, XTE, CESA, etc., were differentially expressed, and primarily downregulated in response to rapamycin and KU63794 (Figure 8, Table S9).

Rapamycin specifically combines with FKBP12 to form a binary complex, which interacts with the FRB domain of TOR to inactivate TOR (Heitman et al., 1991). There are many key and conserved hydrophobic amino acid residues in the drug-binding pocket of the HsFKBP12 for interacting with rapamycin, such as Tyr26, Phe36, Asp37, Phe46, Gln53, Glu54, Val55, Ile56, Trp59, Tyr82, Ile90, Ile91, Phe99 (Van Duyne et al., 1991, 1993; Choi et al., 1996). Alignment of many FKBP12s from representative organisms showed that only residues Phe36, Phe46, Val55, Ile56, Trp59, Tyr82, Ile91, and Phe99 were highly conserved (Figure 2A). The others varied more frequently, even in rapamycin-sensitive yeast, humans, maize, and green algae (Figure 2A). The following five residues are regarded as the most important amino acids due to they establish hydrogen bonds with rapamycin: Asp37, Gln53, Glu54, Ile56, and Tyr82 (Van Duyne et al., 1991, 1993; Choi et al., 1996). However, these amino acids had polymorphisms among the aligned organisms, with the exclusion of Ile56 and Tyr82 (Figure 2A; Agredano-Moreno et al., 2007). In ZmFKBP12 Ser58 is a determinant of FKBP12-rapamycin binding, whereas this residue also exists in the VfFKBP12, which doesn't restore the rapamycin sensitivity of a yeast FKBP12 mutant (Xu et al., 1998). It is difficult to interpret these dual interactions occurring with one or few key amino acids, since the overall 3D structure of proteins play a key role. Therefore, more research needs to be done to decide which residues are essential for forming the ternary complex.

In summary, our results suggest that tomato TOR plays a vital role in controlling seedling growth and tomato FKBP12 is functional in bridging the rapamycin to inactivate SlTOR. The chemical genetic assay using TOR inhibitors indicate that SlFKBP12 could combine the rapamycin to inactivate SlTOR. Furthermore, heterologous expressing SlFKBP12 in *Arabidopsis* could restore rapamycin sensitivity, the protein gel shifting and TOR inhibitors combination assays further demonstrates that SlFKBP12 is functional to bridge the molecular interaction between rapamycin and SlTOR. In addition, comparative expression profiling analysis between rapamycin and KU63794 also support our proposal about the SlFKBP12 and SlTOR in regulating seedling growth. Together, our results will facilitate our understanding of TOR signaling in plant.

## Author contributions

MR, FX, and ZL designed the experiments; FX, PD, ML, KW, FZ, LF, LY, and ZL performed the experiments; MR, FX, PD, and GX analyzed the data; and MR, FX, and PD wrote the manuscript.

### Conflict of interest statement

The authors declare that the research was conducted in the absence of any commercial or financial relationships that could be construed as a potential conflict of interest.

## Supplementary material

Table S1 **Primers used for real-time PCR**.

Table S2 **The targets of TOR inhibitors**.

Table S3 **The homologs of PI3Ks in tomato and other representative organisms**.

Table S4 **The total genes detected in Tomato under the TOR inhibition with TOR inhibitors. (A,B)** The total genes detected in Tomato under the TOR inhibition with rapamycin or KU63794. **(C,D)** The DEGs detected in tomato after the treatment of rapamycin or KU63794. **(E)** The overlapped DEGs detected in tomato both under the treatment of rapamycin and KU63794.

Table S5 **The top 15 up or down-regulated genes in tomato after treating with TOR inhibitors. (A,B)** The top 15 up-regulated genes in tomato after treating with rapamycin. **(C,D)** The top 15 up-regulated genes in tomato after treating with KU63794. Red text indicates the overlapped up-regulated genes and blue shows the overlapped down-regulated genes.

Table S6 **GO enrichment. (A)** The enriched GO terms in tomato under the TOR inhibition with rapamycin. **(B)** The enriched GO terms in tomato under the TOR inhibition with KU63794.

Table S7 **KEGG enrichment. (A)** The KEGG pathway in tomato under rapamycin treatment. **(B)** The KEGG pathway in tomato under KU63794 treatment.

Table S8 **DEGs involved in photosynthesis**.

Table S9 **DEGs involved in cell wall**.

Table S10 **DEGs involved in transporters. (A)** The DEGs related to transporters in tomato after the treatment of rapamycin. **(B)** The DEGs related to transporters in tomato after the treatment of KU63794. **(C)** The overlapped DEGs related to transporters in tomato between the treatment of rapamycin and KU63794.

Figure S1 **Full length of the coding sequence of ***TOR*** gene and predicted protein molecular mass in tomato. (A)** The *SlTOR* were amplified and shown by agarose gel electrophoresis. **(B)** Western blotting shows the SlTOR protein molecular size when compared with AtTOR.

Figure S2 **Rapamycin and asTORis show inhibitory effects on growth of tomato seedlings**. Five DAG seedlings were transferred on 0.5 × MS with rapamycin or asTORis for 5 days. **(A)** The representative seedling phenotype. **(B)** Fresh weight and primary root length relative to control. Values represent mean ± SEM for three biological replicates.

Figure S3 **Rapamycin repress hypocotyl elongation in the dark mediated by FKBP12. (A)** Phenotype of 5DAG seedlings grown on 0.5 MS media with or without rapamycin under dark conditions. **(B)** Corresponding hypocotyl length.

Figure S4 **Gene expression levels both in the treatment of rapamycin and KU63794 compared to control**. The graph shows comparative levels of transcripts of selected genes in the RNA-Seq data and validation by real time-PCR. Solyc00g203660.1, Solyc01g007330.2, Solyc02g077860.1, and Solyc10g047430.1: ribulose-1,5-bisphosphate carboxylase/oxygenase large subunit; Solyc07g055690.1: G-type lectin S-receptor-like serine/threonine-protein kinase At5g35370-like; Solyc01g044250.1: photosystem II 44 kDa protein; Solyc01g007500.2: hypothetical protein M569_00218; Solyc06g009940.1: photosystem I P700 chlorophyll a apoprotein A1; Solyc09g055950.1: photosystem II D2 protein; Solyc04g016170.1: PsaB, partial; Solyc03g113690.1: ABC transporter G family member 23-like; Solyc05g054890.2: ABC transporter G family member 1-like; Solyc06g009280.1: ABC transporter B family member 21-like; Solyc12g006050.1: nitrate transporter 1.3-like; Solyc10g047170.1: sulfate transporter 1; Solyc08g083120.2 and Solyc01g096290.2: ubiquitin extension protein 1; Solyc11g065190.1: ubiquitin-conjugating enzyme E2 19-like; Solyc12g098800.1: extensin-2-like; Solyc03g093080.2: xyloglucan endotransglucosylase/hydrolase protein 23-like; Solyc08g077910.2: expansin-like B1-like.

Figure S5 **The original images corresponding to Figure 5A**.
