# Supplementary material for: Tomato FK506 Binding Protein 12KD (FKBP12) Mediates the Interaction between Rapamycin and Target of Rapamycin (TOR)
Source: Front Plant Sci. 2016 Nov 18;7:1746. doi: 10.3389/fpls.2016.01746 (PMC5114585; doi:10.3389/fpls.2016.01746)
Supplement: Table S1 — Primers used for real-time PCR. [file Table1.DOC]

|  | Primers for qRT-PCR | |
| --- | --- | --- |
| Accession no. | Forward (5’-3’) | Reverse (5’-3’) |
| Solyc07g009380.2 | GCACCGCTACTACATACT | TCTTGTTGCCCATTCCTC |
| Solyc01g099630.2 | ATCCAACCAAGGGCTACCA | TTGACGAACCCAACGAAG |
| Solyc07g055990.2 | GTGACTGGTGAGCCTTATAC | TGTTCCGACCCTTAGCATC |
| Solyc03g093130.2 | TTAGTCAAGGCAAAGGAAAC | TGCAAGCATTAGCACTGAAG |
| Solyc01g087210.2 | CCTTGCCTACTGCACTCT | TTCCTCGATGCTTACTCC |
| Solyc07g005840.2 | TACAGCATAGAGCCACGAGC | AGTCCTCCACTTTGTCCCA |
| Solyc02g072240.2 | AGAGGACTGGTGGCGTAAC | AGCACAGACCATAGCACAAC |
| Solyc03g093390.2 | TGTTCAGGCAAGGGAGTAAG | ATTCTATCACAACCGCAAAG |
| Solyc12g006340.1 | GGTATCTACTTCTGGCTTCA | CTGCTCACTATGCCCTTG |
| Solyc05g047460.2 | TGGCTTCCGATTATGTCAC | TGTCCTCCAGTTGTCCCTCA |
| Solyc01g080460.2 | GACTGTTAGACCCACCAC | GGAAATACTGAAATACCCT |
| Solyc04g076880.2 | TCCACCAGTCAGCAAATTG | CTCCAGCCCAAATACCTC |
| Solyc10g084410.1 | CGGAGCCATCAATATCAG | TGGAAATGCCTTCTTACT |
| Solyc11g005820.1 | AAGCCTCAAAGCAGACAC | TCCATCTTCCCTAATAGCA |
| Solyc03g113040.2 | TCCCTTGTTACAGATACCG | TAGGAGAACACTGCGATTG |
| Solyc03g113690.1 | ACCTATTCAGCTCAACCCTC | GTCCAAATCCTCCCACAAG |
| Solyc07g032490.2 | CAGTGACCAGGGTTGAGG | TCCCTTTGGGTGATTGTG |
| Solyc10g024490.1 | TACAGGCAACCCTCTATCA | ACGCCATTTGAGTAAACACA |

**Supplementary Table S1. Primers used for real-time PCR**
