# Supplementary material for: Tomato FK506 Binding Protein 12KD (FKBP12) Mediates the Interaction between Rapamycin and Target of Rapamycin (TOR)
Source: Front Plant Sci. 2016 Nov 18;7:1746. doi: 10.3389/fpls.2016.01746 (PMC5114585; doi:10.3389/fpls.2016.01746)
Supplement: Table S2 — The targets of TOR inhibitors. [file Table2.DOC]

**The targets of TOR inhibitors**

| **Inhibitor Name** | **mTOR** | **mTORC1** | **mTORC2** | **Other Targets** |
| --- | --- | --- | --- | --- |
| [**Rapamycin (Sirolimus)**](http://www.selleck.cn/products/Rapamycin.html) | **++++**  (~0.1 nM) |  |  |  |
| [**AZD8055**](http://www.selleck.cn/products/AZD8055.html) | **++++**  (0.8 nM) |  |  | **DNA-PK (**1,370 nM**),**  **PI3Kδ (**3,200 nM**),**  **PI3Kα (**3,590 nM**)** |
| [**KU63794**](http://www.selleck.cn/products/KU-0063794.html) |  | **++**  **(**~10 nM**)** | **++**  **(**~10 nM**)** |  |
| [**Torin 1**](http://www.selleck.cn/products/torin-1.html) | **+++**  **(**4.32 nM**)** | **+++**  **(**2 nM**)** | **++**  **(**10 nM**)** | **DNA-PK (**6.34 nM**),**  **PI3Kα (**250 nM**)**  **PI3Kδ (**564 nM**)**  **PI3Kγ (**171 nM**),**  **C2α (**176 nM**)** |

**Notes:**

1. These data come from Selleckchem.com (<http://www.selleckchem.com/>). The details about the IC50 of each TOR inhibitor are shown in the bracket (please click the link of the agent on <http://www.selleckchem.com/>).
2. “+” represents the strength of the inhibitory effects. The more “+”, the stronger inhibition.
