# Supplementary material for: Tomato FK506 Binding Protein 12KD (FKBP12) Mediates the Interaction between Rapamycin and Target of Rapamycin (TOR)
Source: Front Plant Sci. 2016 Nov 18;7:1746. doi: 10.3389/fpls.2016.01746 (PMC5114585; doi:10.3389/fpls.2016.01746)
Supplement: Table S3 — The homologs of PI3Ks in tomato and other representative organisms. [file Table3.DOC]

**Supplemental Table S3 The homologs of PI3Ks in tomato and other representative organisms.**

| Subunits  Species | DNA-PK | PI3Kγ | C2α | PI3Kδ | PI3Kα | ATM | ATR |
| --- | --- | --- | --- | --- | --- | --- | --- |
| *Mus musculus* | 1 | 3 | 1 | 5 | 1 | 1 | 1 |
| *Homo sapiens* | 1 | 4 | 1 | 1 | 1 | 1 | 1 |
| *Arabidopsis thaliana* | - | - | - | - | - | 1 | 1 |
| *Solanum lycopersicum* | - | - | - | - | - | 1 | 1 |

# Note: DNA-PK, DNA-dependent protein kinase; PI3K, phosphoinositide 3-kinase; ATM, Ataxia telangiectasia-mutated; ATR, Ataxia Telangiectasia-Mutated and Rad3-Related.
