# Supplementary material for: Tomato FK506 Binding Protein 12KD (FKBP12) Mediates the Interaction between Rapamycin and Target of Rapamycin (TOR)
Source: Front Plant Sci. 2016 Nov 18;7:1746. doi: 10.3389/fpls.2016.01746 (PMC5114585; doi:10.3389/fpls.2016.01746)
Supplement: Table S8 — DEGs involved in photosynthesis. [file Table8.DOC]

Supplementary Table S8 The DEGs related to photosynthesis in Tomato under the condition of TOR inhibition with rapamycin and KU63794,.

| Gene ID | Putative function | Log_2_FC | ^a^FDR | ^b^Log_2_FC | ^b^FDR |
| --- | --- | --- | --- | --- | --- |
|  | **Light reaction** |  |  |  |  |
| Solyc00g230080.1 | Photosystem II D2 protein | -2.13 | 0.02 | -2.17 | 0.00 |
| Solyc01g007520.2 | Photosystem II protein H | -2.02 | 0.01 | -2.33 | 0.00 |
| Solyc01g017300.1 | Photosystem I P700 apoprotein A1 | -3.72 | 3.30E-06 | -3.06 | 6.48E-06 |
| Solyc01g017320.1 | Photosystem1 subunit A | -3.42 | 0.00 | -2.97 | 6.58E-05 |
| Solyc01g017330.1 | Photosystem I P700 apoprotein A1 | -3.86 | 4.36E-06 | -2.86 | 4.43E-06 |
| Solyc01g018090.1 | Photosystem I P700 apoprotein A1 | -3.57 | 0.00 | -3.29 | 4.52E-05 |
| Solyc01g044250.1 | Photosystem II 44 kDa protein | -2.35 | 0.04 | -2.11 | 0.02 |
| Solyc02g011990.1 | Photosystem II 32 kDa protein | 1.57 | 0.04 |  |  |
| Solyc02g036320.1 | Photosystem I P700 chlorophyll a apoprotein A2 | -3.36 | 1.63E-06 | -2.34 | 3.65E-05 |
| Solyc05g020010.1 | Photosystem I P700 apoprotein A2 | -3.77 | 2.47E-07 | -2.48 | 8.46E-06 |
| Solyc06g009940.1 | Photosystem I P700 chlorophyll a apoprotein A1 ` | -4.03 | 2.25E-07 | -2.49 | 2.31E-06 |
| Solyc07g021230.1 | Photosystem I P700 chlorophyll a apoprotein A2 | -3.92 | 0.01 | -3.70 | 0.00 |
| Solyc07g021240.1 | Photosystem I P700 apoprotein A2 (chloroplast) | -3.41 | 1.39E-05 | -2.35 | 0.00 |
| Solyc09g015300.1 | Photosystem I P700 apoprotein A1 | -3.67 | 0.01 | -3.29 | 0.00 |
| Solyc09g055950.1 | Photosystem II D2 protein | -2.76 | 0.00 | -1.90 | 0.00 |
| Solyc09g059640.1 | Photosystem I P700 chlorophyll a apoprotein A2 | -3.20 | 2.09E-05 | -2.31 | 7.51E-05 |
| Solyc09g064580.2 | Photosystem II reaction center protein M (chloroplast) | 5.78 | 0.00 | 5.21 | 0.01 |
| Solyc10g017900.1 | Photosystem I P700 chlorophyll a apoprotein A1 | -3.88 | 2.67E-06 | -2.44 | 3.46E-05 |
| Solyc10g017910.1 | Photosystem I P700 apoprotein A2 | -3.16 | 0.00 | -2.97 | 0.00 |
| Solyc10g052740.1 | Photosystem I P700 chlorophyll a apoprotein A1-like | -4.04 | 5.86E-08 | -2.72 | 5.95E-07 |
| Solyc11g013780.1 | Photosystem II 32 kDa protein | 2.02 | 0.01 |  |  |
| Solyc11g056340.1 | Photosystem II D1 protein | 1.76 | 0.02 |  |  |
| Solyc12g032960.1 | Photosystem II 32 kDa protein | -3.72 | 3.80E-08 | -2.81 | 2.83E-07 |
| Solyc12g032990.1 | Photosystem I P700 apoprotein A2 | -3.52 | 0.00 | -2.64 | 0.00 |
| Solyc12g033000.1 | Photosystem I P700 apoprotein A2 | -3.15 | 0.01 | -2.26 | 0.01 |
| Solyc12g033040.1 | Photosystem I P700 apoprotein A1 | -3.75 | 1.66E-05 | -2.88 | 2.88E-06 |
| Solyc12g033050.1 | Photosystem I P700 chlorophyll a apoprotein A2 | -3.05 | 0.00 | -2.48 | 0.00 |
| Solyc12g033060.1 | Photosystem I P700 chlorophyll a apoprotein A2 | -3.23 | 1.52E-10 | -2.63 | 1.16E-09 |
| Solyc12g039030.1 | Photosystem II protein D1 | 1.67 | 0.01 |  |  |
| Solyc12g062600.1 | Photosystem I P700 apoprotein A2 | -3.70 | 0.00 | -2.69 | 0.00 |
| Solyc06g009950.1 | PSI P700 apoprotein A2 | -2.97 | 0.00 | -3.35 | 9.08E-06 |
| Solyc01g017740.1 | Cytochrome b6 | -3.31 | 0.04 | -3.04 | 0.01 |
| Solyc01g007540.2 | Cytochrome b6/f complex subunit IV | -3.30 | 0.00 | -2.04 | 0.01 |
| Solyc01g007530.2 | Cytochrome b6 | -3.25 | 2.57E-07 | -2.53 | 1.08E-06 |
| Solyc10g006530.2 | psbQ-like protein 2, chloroplastic-like | 1.78 | 1.64E-06 | 1.54 | 0.00 |
| Solyc02g020960.1 | Photosystem I P700 chlorophyll a apoprotein A1 | -3.75 | 0.00 | -3.63 | 0.00 |
| Solyc10g017890.1 | Photosystem I P700 chlorophyll a apoprotein | -3.61 | 9.69E-10 | -2.90 | 1.95E-09 |
| Solyc01g007380.1 | Cytochrome f | -1.94 | 0.03 |  |  |
| Solyc01g007500.2 | Photosystem II CP47 chlorophyll apoprotein | -3.23 | 0.00 | -2.56 | 0.00 |
| Solyc04g016170.1 | Photosystem I P700 chlorophyll a apoprotein A2 | -4.41 | 5.94E-08 | -3.18 | 3.80E-08 |
| Solyc07g054290.1 | Photosystem II family protein | 1.18 | 0.00 | 1.04 | 0.00 |
| Solyc12g032980.1 | Photosystem I P700 chlorophyll a apoprotein A2 | -2.78 | 2.09E-05 | -2.69 | 1.67E-06 |
| Solyc12g035730.1 | Photosystem I P700 chlorophyll a apoprotein A2 |  |  | -2.70 | 0.04 |
| Solyc10g047410.1 | Photosystem II CP43 chlorophyll apoprotein |  |  | -1.73 | 0.02 |
| Solyc06g069730.2 | Chlorophyll a-b binding protein 4 |  |  | 1.49 | 1.73E-05 |
| Solyc09g015340.1 | Photosystem I P700 chlorophyll a apoprotein A1 |  |  | -3.45 | 0.02 |
| Solyc09g015340.1 | Photosystem I P700 chlorophyll a apoprotein A1 |  |  | -2.75 | 0.02 |
|  | **Carbon fixation** |  |  |  |  |
| Solyc00g203660.1 | Ribulose-1,5-bisphosphate carboxylase/oxygenase large subunit | -3.87 | 0.03 | -3.40 | 0.00 |
| Solyc02g077860.1 | Ribulose-1,5-bisphosphate carboxylase/oxygenase large subunit | -3.32 | 0.04 | -2.93 | 0.01 |
| Solyc01g007330.2 | Ribulose-1,5-bisphosphate carboxylase/oxygenase large subunit | -2.93 | 2.07E-12 | -2.62 | 2.60E-11 |
| Solyc10g047430.1 | Ribulose-1,5-bisphosphate carboxylase/oxygenase large subunit | -2.54 | 0.00 | -2.17 | 9.60E-05 |
| Solyc06g063090.2 | Alanine aminotransferase 2, mitochondrial-like | -1.30 | 0.00 |  |  |
| Solyc05g050120.2 | Cytosolic NADP-malic enzyme | -1.17 | 0.03 |  |  |
| Solyc12g088160.1 | Phosphoenolpyruvate carboxykinase | -1.38 | 0.03 | -1.65 | 0.01 |
| Solyc04g076880.2 | Phosphoenolpyruvate carboxykinase | -1.39 | 0.00 | -1.65 | 0.00 |
| Solyc08g066360.2 | NADP-dependent malic enzyme-like | 1.02 | 0.01 |  |  |
| Solyc01g080460.2 | Pyruvate, phosphate dikinase, chloroplastic-like | -1.93 | 1.05E-08 | -1.72 | 6.29E-07 |
| Solyc07g021200.1 | Ribulose-1,5-bisphosphate carboxylase/oxygenase large subunit |  |  | -3.13 | 0.00 |
| Solyc08g068330.2 | Aspartate aminotransferase, chloroplastic-like |  |  | -1.14 | 0.00 |

“a” repesents the data from rapamycin; “b” represents the data from KU.
