# Supplementary material for: Tomato FK506 Binding Protein 12KD (FKBP12) Mediates the Interaction between Rapamycin and Target of Rapamycin (TOR)
Source: Front Plant Sci. 2016 Nov 18;7:1746. doi: 10.3389/fpls.2016.01746 (PMC5114585; doi:10.3389/fpls.2016.01746)
Supplement: Table S9 — DEGs involved in cell wall. [file Table9.DOC]

**Supplementary Table S9. Differentially expressed genes related to cell wall expansin**

| Gene ID | Putative function | ^a^Log_2_FC | ^a^FDR | ^b^Log_2_FC | ^b^FDR |
| --- | --- | --- | --- | --- | --- |
|  | **Expansin** |  |  |  |  |
| Solyc02g081210.2 | Expansin-A13-like | 1.01 | 0.04 | 1.13 | 0.02 |
| Solyc06g076220.2 | Expansin18 precursor | 1.41 | 1.85E-05 | 1.40 | 5.61E-05 |
| Solyc08g077330.2 | Expansin-like B1-like | -1.72 | 2.86E-07 | -1.68 | 9.10E-07 |
| Solyc08g077900.2 | Expansin-like B1-like | -2.48 | 5.35E-13 | -2.05 | 5.17E-08 |
| Solyc08g077910.2 | Expansin-like B1-like | -1.81 | 0.001 | -3.21 | 7.08E-07 |
| Solyc03g093390.2 | Beta expansin precursor |  |  | -1.42 | 0.01 |
| Solyc07g054170.2 | Expansin-B3-like |  |  | 1.40 | 0.14 |
|  | **Extensin** |  |  |  |  |
| Solyc03g082790.2 | Extensin-like protein Dif54 precursor | -1.03 | 0.01 |  |  |
| Solyc04g071100.1 | Extensin-3-like | -1.79 | 0.01 | -2.03 | 0.00 |
| Solyc12g098800.1 | Extensin-2-like | -5.00 | 0.03 | -5.00 | 0.03 |
| Solyc02g078100.2 | Pistil-specific extensin-like protein-like |  |  | 1.82 | 0.00 |
| Solyc09g082530.1 | Leucine-rich repeat extensin-like protein 3-like |  |  | -2.24 | 0.03 |
|  | **Xyloglucan endotransglucosylase/hydrolase (XTE/XTH) protein** |  |  |  |  |
| Solyc01g005120.2 | XTE/XTH protein 28-like | 1.27 | 0.01 |  |  |
| Solyc01g080010.2 | Xyloglucan-specific fungal endoglucanase inhibitor protein precursor | -2.00 | 1.68E-10 | -1.63 | 6.30E-07 |
| Solyc02g080160.2 | XTE/XTH protein 8-like | 1.47 | 0.01 | 1.52 | 0.01 |
| Solyc03g093080.2 | XTE/XTH protein 23-like | -3.10 | 0.00 | -2.55 | 0.00 |
| Solyc03g093120.2 | XTE/XTH protein 23-like | -2.65 | 0.01 | -2.54 | 0.00 |
| Solyc03g093130.2 | XTE/XTH3 precursor | -2.41 | 0.00 | -2.41 | 0.00 |
| Solyc05g046290.2 | XTE/XTH protein 23-like | -1.41 | 6.39E-05 | -1.53 | 4.26E-05 |
| Solyc07g009380.2 | XTE LeXET2 precursor | -1.16 | 0.05 |  |  |
| Solyc07g052980.2 | XTE/XTH protein 9 | 1.16 | 0.00 | 1.01 | 0.01 |
| Solyc07g055990.2 | XTE/XTH protein 16-like | -3.32 | 4.32E-06 | -2.81 | 1.72E-05 |
| Solyc07g056000.2 | XTE precursor | -2.49 | 0.03 | -2.21 | 0.02 |
| Solyc11g066270.1 | XTE/XTH 6 precursor | -1.47 | 0.00 | -1.97 | 7.66E-05 |
| Solyc01g081060.2 | XTE/XTH 5 precursor |  |  | -1.02 | 0.00 |
| Solyc03g093110.2 | XTE/XTH protein 23-like |  |  | -2.31 | 0.01 |
|  | **Cellulose synthase A (CESA)** |  |  |  |  |
| Solyc02g072240.2 | Cellulose synthase A catalytic subunit 8 | -1.84 | 0.00 | -1.56 | 0.00 |
| Solyc07g005840.2 | Cellulose synthase A catalytic subunit 7 | -1.23 | 0.02 | -1.52 | 0.00 |
| Solyc07g065660.2 | Cellulose synthase-like protein E1-like | -1.24 | 0.03 |  |  |
| Solyc09g072820.2 | Cellulose synthase A catalytic subunit 4 | -1.67 | 0.00 | -1.40 | 0.01 |
| Solyc07g051820.2 | Cellulose synthase-like protein B3-like |  |  | -1.37 | 0.03 |
|  | **Pectate lyase** |  |  |  |  |
| Solyc05g014000.2 | Pectate lyase 5-like | -1.02 | 0.02 |  |  |
| Solyc03g071570.2 | Pectate lyase 13-like | 1.20 | 0.03 | 1.36 | 0.01 |
| Solyc09g008380.2 | Pectate lyase 12-like | 1.58 | 0.00 | 1.61 | 0.00 |
|  | **Pectinesterase** |  |  |  |  |
| Solyc01g098940.2 | Pectinesterase/pectinesterase inhibitor 25-like | 1.81 | 0.02 |  |  |
| Solyc02g075620.2 | Pectinesterase 53-like | 1.16 | 0.01 | 1.11 | 0.01 |
| Solyc03g083870.2 | pectinesterase/pectinesterase inhibitor 17-like | -2.38 | 0.00 | -3.03 | 0.00 |
| Solyc08g078640.1 | Pectinesterase 68-like | 1.88 | 5.89E-07 | 1.89 | 1.02E-06 |
| Solyc09g075350.2 | Pectinesterase/pectinesterase inhibitor 40-like | 1.22 | 0.02 | 1.32 | 0.01 |
| Solyc07g043240.2 | Pectinesterase 8-like |  |  | -4.91 | 0.00 |
|  | **Arabinogalactan** |  |  |  |  |
| Solyc11g010390.1 | Classical arabinogalactan protein 4-like | -1.06 | 0.04 |  |  |
| Solyc08g008230.2 | Arabinogalactan peptide 22-like | 1.06 | 0.00 |  |  |
| Solyc10g078580.1 | Arabinogalactan peptide 14-like | 1.66 | 1.97E-06 | 1.44 | 0.00 |
| Solyc10g011730.2 | Arabinogalactan peptide 20-like | 1.71 | 0.03 | 1.68 | 0.03 |
|  | **Proline-rich protein** |  |  |  |  |
| Solyc02g089620.2 | Proline dehydrogenase 2 | 1.47 | 9.70E-07 |  |  |
| Solyc07g007860.1 | 36.4 kDa proline-rich protein-like | 1.99 | 0.00 | 1.76 | 0.01 |
| Solyc08g074480.1 | 14 kDa proline-rich protein DC2.15-like | 1.14 | 0.00 |  |  |
| Solyc08g078940.1 | 14 kDa proline-rich protein DC2.15-like | 2.39 | 4.67E-07 | 2.46 | 4.25E-07 |
| Solyc09g072770.1 | Repetitive proline-rich cell wall protein 2-like | -1.95 | 1.53E-07 | -2.04 | 4.85E-08 |
| Solyc03g096390.2 | Proline transporter 1 |  |  | -1.01 | 0.01 |
| Solyc10g054900.1 | Proline-rich protein 4-like |  |  | -3.19 | 0.03 |
|  | **Glycoprotein** |  |  |  |  |
| Solyc02g076830.1 | Epidermis-specific secreted glycoprotein EP1-like | -2.77 | 0.00 | -1.87 | 0.01 |
| Solyc07g062480.1 | Epidermis-specific secreted glycoprotein EP1-like | -1.83 | 6.22E-07 | -1.03 | 0.00 |
|  | **Glycine, alanine and asparagine-rich protein** |  |  |  |  |
| Solyc04g014300.1 | glycine, alanine and asparagine-rich protein-like | -4.55 | 0.04 |  |  |
| Solyc04g014310.1 | glycine, alanine and asparagine-rich protein-like | -3.79 | 0.01 | -3.21 | 0.00 |
| Solyc04g014350.1 | glycine, alanine and asparagine-rich protein-like | -3.47 | 0.00 | -2.38 | 0.00 |
|  | **Glycine-rich protein** |  |  |  |  |
| Solyc01g014280.2 | glycine-rich cell wall structural protein 1.8-like | -2.68 | 0.00 | -2.21 | 0.00 |
| Solyc06g061200.1 | glycine-rich protein TomR2 | -1.73 | 8.73E-08 | -1.31 | 0.00 |
| Solyc10g051380.1 | glycine-rich RNA-binding protein-like |  |  | 1.01 | 0.00 |

“a” repesents the data from rapamycin VS DMSO; “b” represents the data from KU VS DMSO.
