# Supplementary figures and images for: Tomato FK506 Binding Protein 12KD (FKBP12) Mediates the Interaction between Rapamycin and Target of Rapamycin (TOR)
Source: Front Plant Sci. 2016 Nov 18;7:1746. doi: 10.3389/fpls.2016.01746 (PMC5114585; doi:10.3389/fpls.2016.01746)

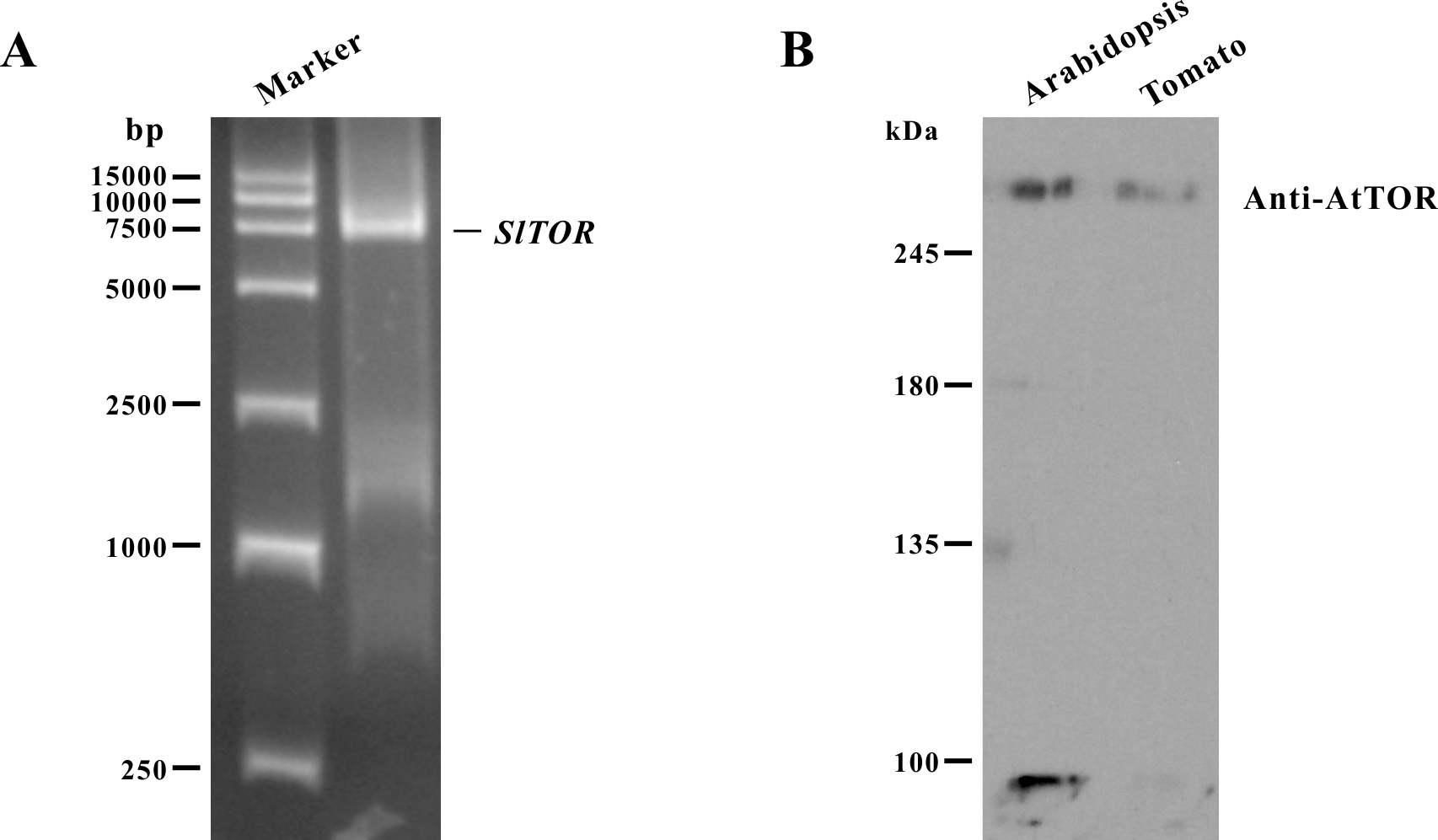

Supplement: Figure S1 — Full length of the coding sequence of TOR gene and predicted protein molecular mass in tomato. (A) The SlTOR were amplified and shown by agarose gel electrophoresis. (B) Western blotting shows the SlTOR protein molecular size when compared with AtTOR. [file Image1.JPEG]

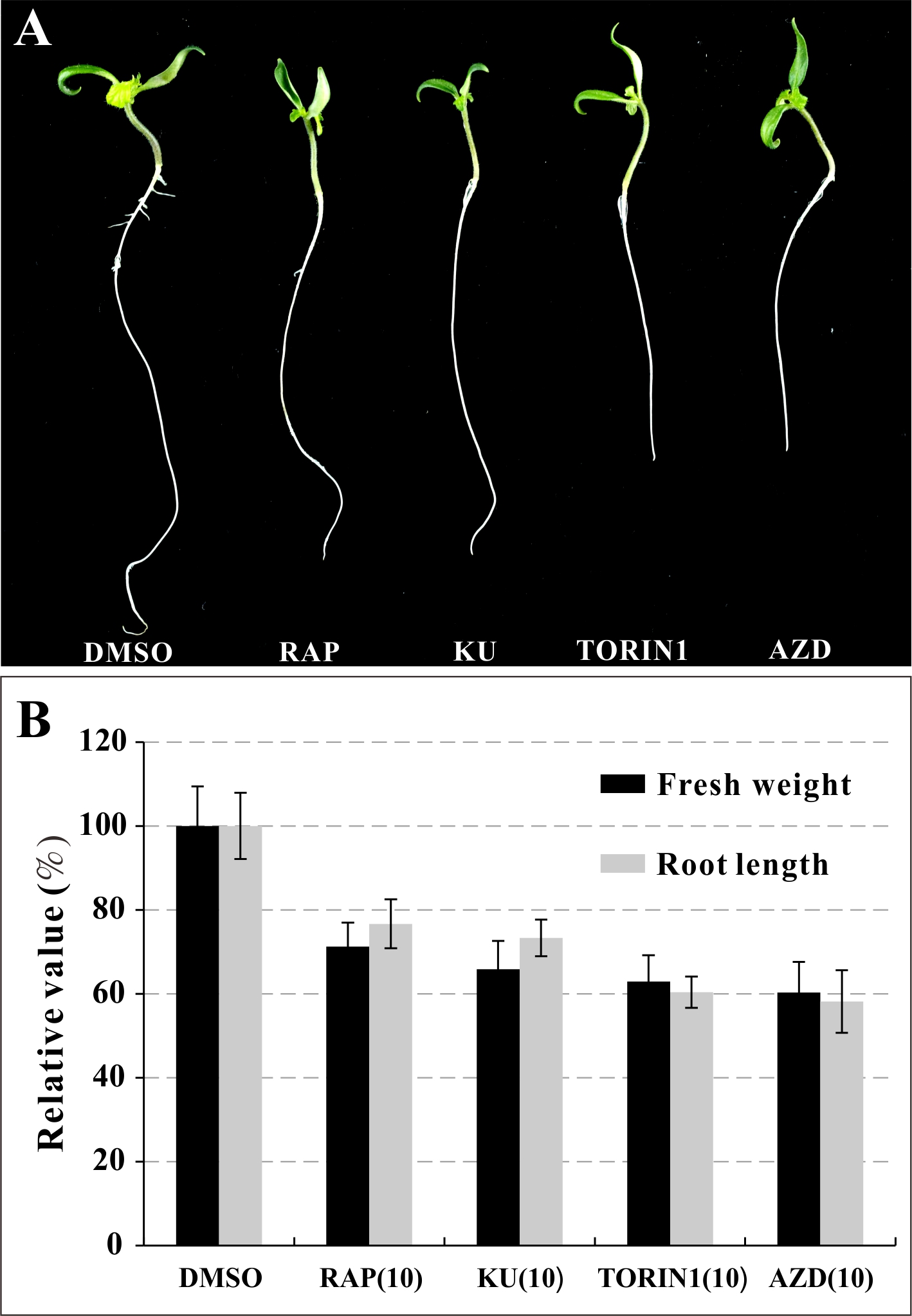

Supplement: Figure S2 — Rapamycin and asTORis show inhibitory effects on growth of tomato seedlings. Five DAG seedlings were transferred on 0.5 × MS with rapamycin or asTORis for 5 days. (A) The representative seedling phenotype. (B) Fresh weight and primary root length relative to control. Values represent mean ± SEM for three biological replicates. [file Image2.JPEG]

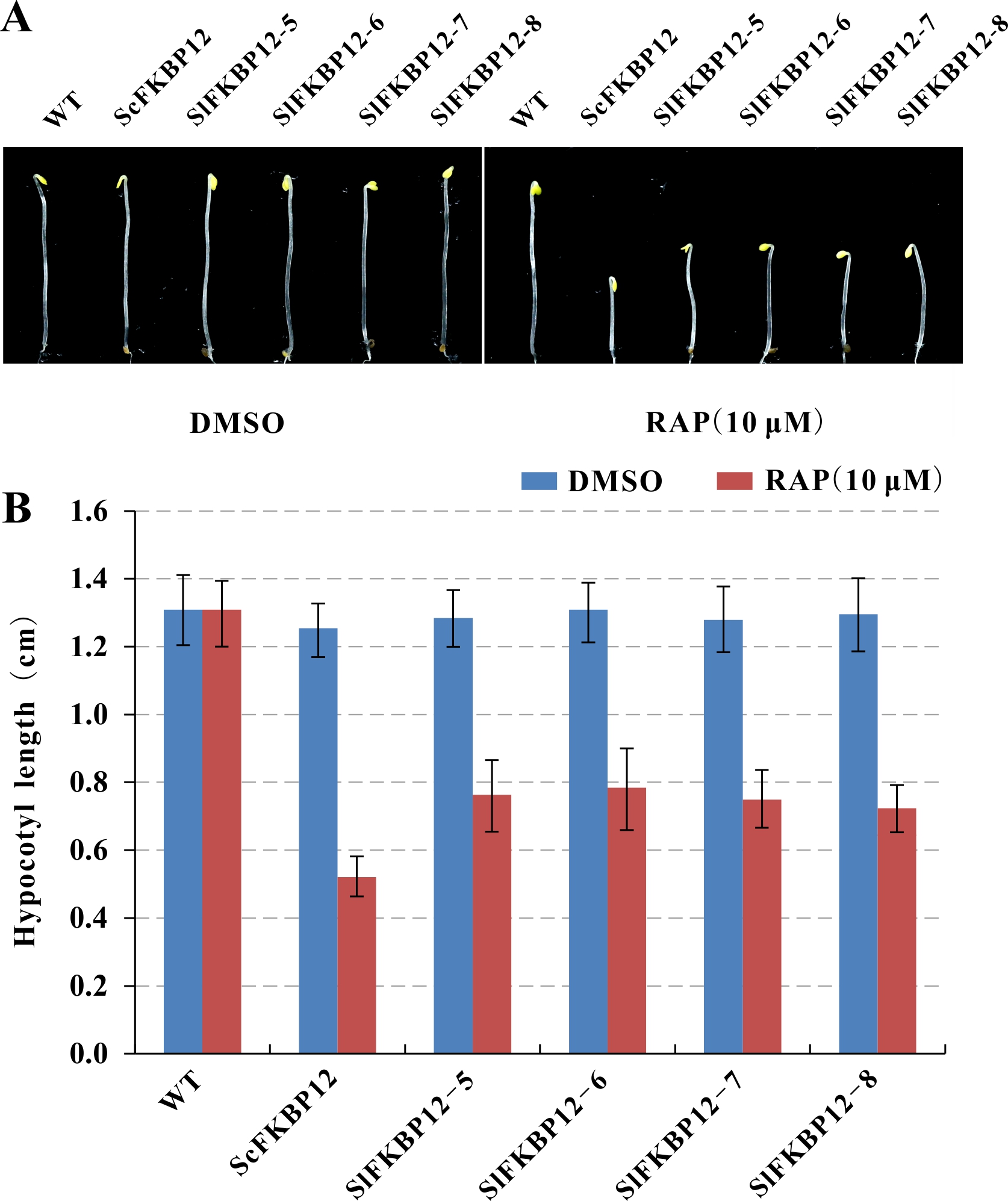

Supplement: Figure S3 — Rapamycin repress hypocotyl elongation in the dark mediated by FKBP12. (A) Phenotype of 5DAG seedlings grown on 0.5 MS media with or without rapamycin under dark conditions. (B) Corresponding hypocotyl length. [file Image3.JPEG]

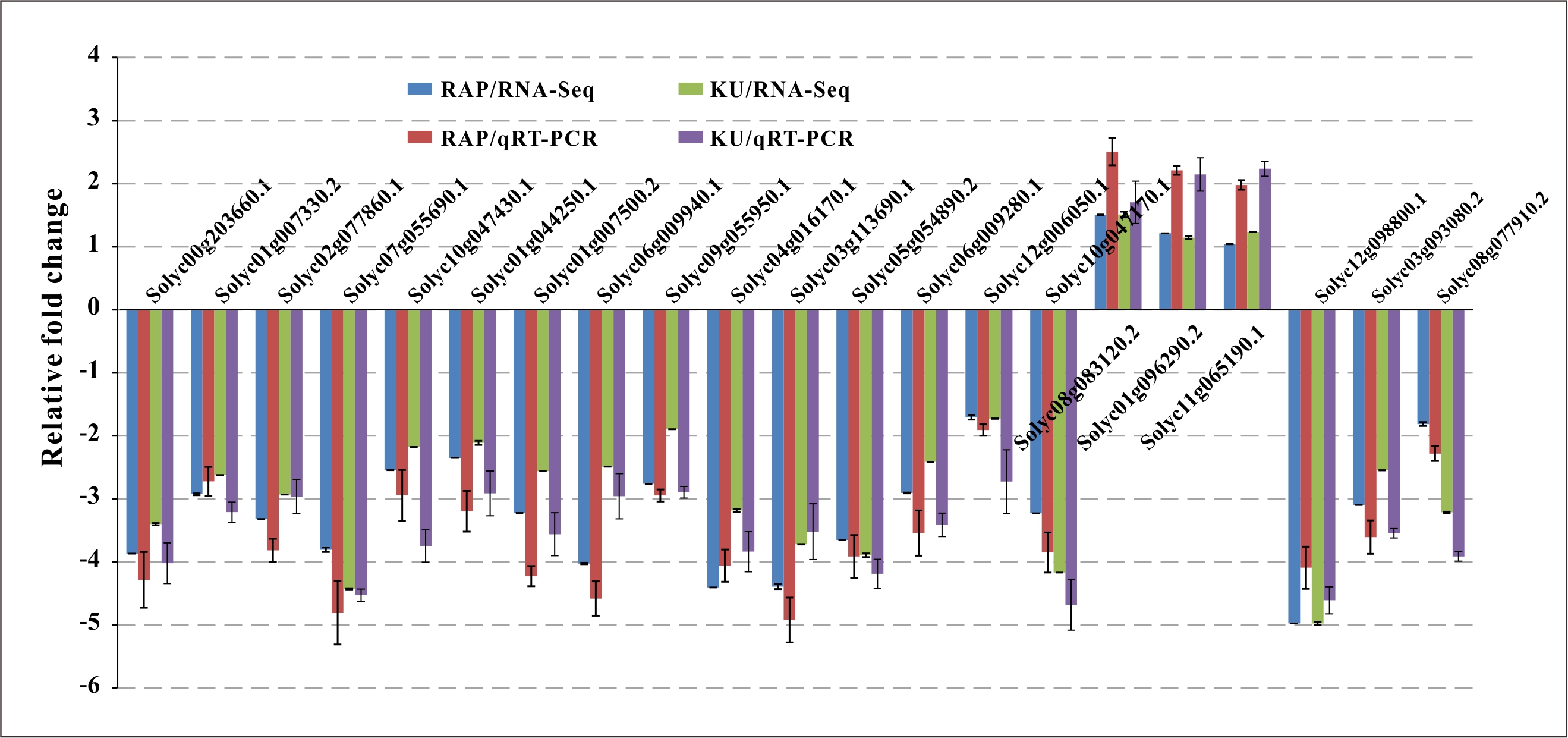

Supplement: Figure S4 — Gene expression levels both in the treatment of rapamycin and KU63794 compared to control. The graph shows comparative levels of transcripts of selected genes in the RNA-Seq data and validation by real time-PCR. Solyc00g203660.1, Solyc01g007330.2, Solyc02g077860.1, and Solyc10g047430.1: ribulose-1,5-bisphosphate carboxylase/oxygenase large subunit; Solyc07g055690.1: G-type lectin S-receptor-like serine/threonine-protein kinase At5g35370-like; Solyc01g044250.1: photosystem II 44 kDa protein; Solyc01g007500.2: hypothetical protein M569_00218; Solyc06g009940.1: photosystem I P700 chlorophyll a apoprotein A1; Solyc09g055950.1: photosystem II D2 protein; Solyc04g016170.1: PsaB, partial; Solyc03g113690.1: ABC transporter G family member 23-like; Solyc05g054890.2: ABC transporter G family member 1-like; Solyc06g009280.1: ABC transporter B family member 21-like; Solyc12g006050.1: nitrate transporter 1.3-like; Solyc10g047170.1: sulfate transporter 1; Solyc08g083120.2 and Solyc01g096290.2: ubiquitin extension protein 1; Solyc11g065190.1: ubiquitin-conjugating enzyme E2 19-like; Solyc12g098800.1: extensin-2-like; Solyc03g093080.2: xyloglucan endotransglucosylase/hydrolase protein 23-like; Solyc08g077910.2: expansin-like B1-like. [file Image4.JPEG]

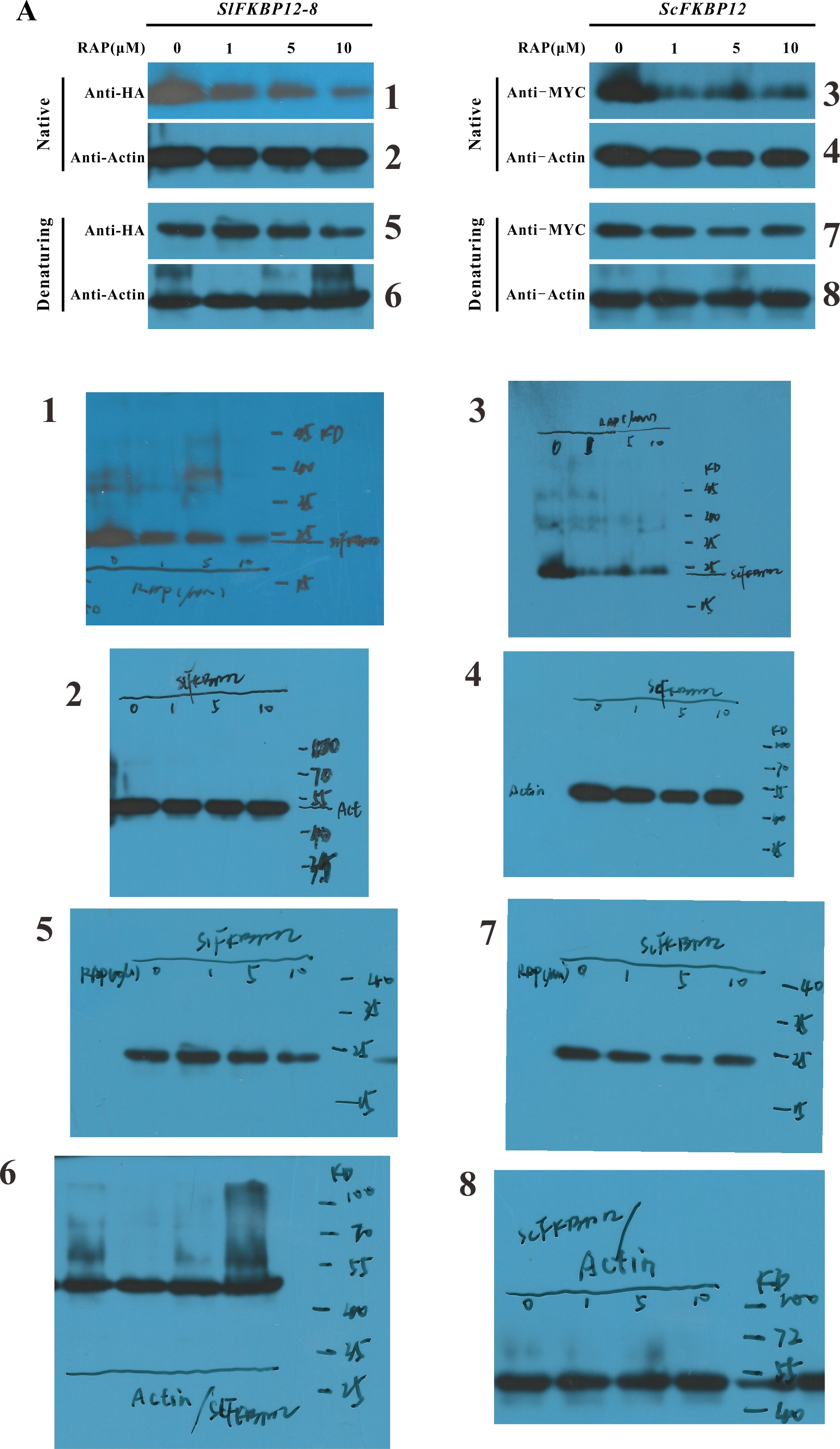

Supplement: Figure S5 — The original images corresponding to Figure 5A. [file Image5.JPEG]
